# Distinct pathogenic roles for resident and monocyte-derived macrophages in lupus nephritis

**DOI:** 10.1172/jci.insight.159751

**Published:** 2022-11-08

**Authors:** Nathan Richoz, Zewen K. Tuong, Kevin W. Loudon, Eduardo Patiño-Martínez, John R. Ferdinand, Anaïs Portet, Kathleen R. Bashant, Emeline Thevenon, Francesca Rucci, Thomas Hoyler, Tobias Junt, Mariana J. Kaplan, Richard M. Siegel, Menna R. Clatworthy

**Affiliations:** 1 Molecular Immunity Unit, University of Cambridge Department of Medicine, Medical Research Council Laboratory of Molecular Biology, Cambridge, United Kingdom.; 2 Cambridge Institute of Therapeutic Immunology and Infectious Diseases, Department of Medicine, University of Cambridge School of Clinical Medicine, United Kingdom.; 3 National Institute of Arthritis and Musculoskeletal and Skin Diseases, National Institutes of Health, Bethesda, Maryland, USA.; 4 Cellular Genetics programme, Wellcome Sanger Institute, Hinxton, United Kingdom.; 5 Novartis Institutes for BioMedical Research, Basel, Switzerland.

**Keywords:** Autoimmunity, Immunology, Lupus, Macrophages

## Abstract

Lupus nephritis is a serious complication of systemic lupus erythematosus, mediated by IgG immune complex (IC) deposition in kidneys, with limited treatment options. Kidney macrophages are critical tissue sentinels that express IgG-binding Fcγ receptors (FcγRs), with previous studies identifying prenatally seeded resident macrophages as major IC responders. Using single-cell transcriptomic and spatial analyses in murine and human lupus nephritis, we sought to understand macrophage heterogeneity and subset-specific contributions in disease. In lupus nephritis, the cell fate trajectories of tissue-resident (TrMac) and monocyte-derived (MoMac) kidney macrophages were perturbed, with disease-associated transcriptional states indicating distinct pathogenic roles for TrMac and MoMac subsets. Lupus nephritis–associated MoMac subsets showed marked induction of FcγR response genes, avidly internalized circulating ICs, and presented IC-opsonized antigen. In contrast, lupus nephritis–associated TrMac subsets demonstrated limited IC uptake, but expressed monocyte chemoattractants, and their depletion attenuated monocyte recruitment to the kidney. TrMacs also produced B cell tissue niche factors, suggesting a role in supporting autoantibody-producing lymphoid aggregates. Extensive similarities were observed with human kidney macrophages, revealing cross-species transcriptional disruption in lupus nephritis. Overall, our study suggests a division of labor in the kidney macrophage response in lupus nephritis, with treatment implications — TrMacs orchestrate leukocyte recruitment while MoMacs take up and present IC antigen.

## Graphical abstract

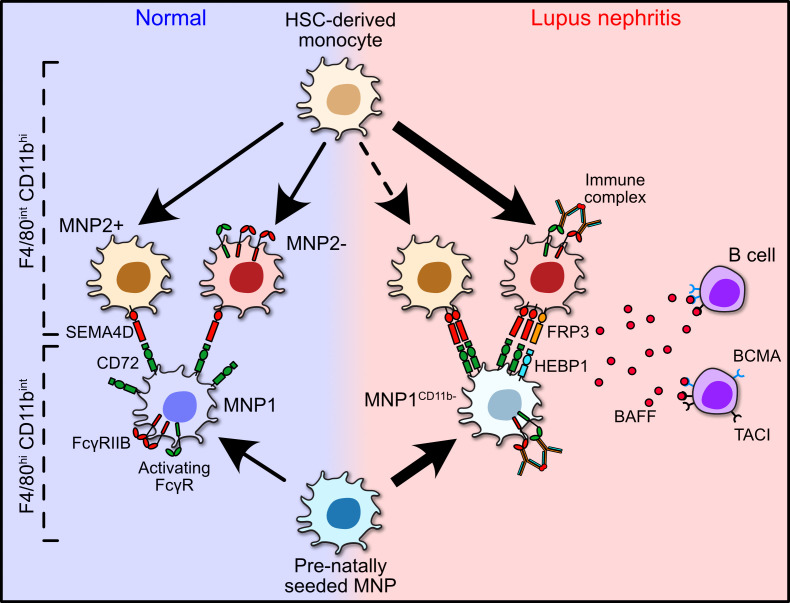

## Introduction

All tissues contain a network of mononuclear phagocytes (MNPs), including macrophages and dendritic cells (DCs) (1), tissue sentinels that express a range of receptors to enable pathogen recognition, including IgG-opsonized microbes. However, this capacity may also promote deleterious inflammation and tissue damage in the context of autoimmunity, for example, by binding autoreactive IgG immune complexes (ICs) in patients with systemic lupus erythematosus (SLE). One of the most serious manifestations of SLE is lupus nephritis, a condition characterized by IgG IC deposition in the kidney, activating complement and local immune cells, including macrophages, by engaging Fcγ receptors (FcγRs) (2–4). Macrophage IgG IC stimulation results in the generation of proinflammatory cytokines and mediators (5, 6); therefore unsurprisingly, *FCGR* polymorphisms that augment FcγR-mediated macrophage activation are associated with susceptibility to SLE and lupus nephritis (5, 7). In established lupus nephritis, B/plasma cell aggregates capable of local autoantibody production have also been described (8, 9). Current treatments for SLE largely consist of nonspecific immunosuppressants, with blockade of the B cell survival cytokine, B cell activating factor of the TNF receptor family (BAFF), the only new therapeutic option (10). A better understanding of cell type–specific responses in lupus nephritis is needed to identify new and more targeted treatments.

Tissue macrophages are seeded into organs prenatally from yolk sac or fetal liver progenitors and are variably replaced postnatally by monocyte precursors (11–13). Early fate mapping and parabiosis studies suggested that in adult mouse kidney around half of macrophages are prenatally seeded tissue-resident cells (TrMacs), do not circulate, and express high levels of F4/80, while monocyte-derived kidney macrophages (MoMacs) have low F4/80 expression and are CD11b high (11). More recent parabiosis experiments confirmed little exchange of F4/80^hi^ cells (14, 15), though kidney injury or pathology may influence the extent to which renal macrophages are replenished from different precursors (16, 17).

Macrophages adopt tissue-specific transcriptional profiles within organs (18). Recent single-cell RNA sequencing (scRNA-Seq) studies showed that MoMacs within lung, heart, liver, and skin progress along 2 distinct cell fates, occupying different anatomical niches within the tissues (19); Lyve1^lo^MHCII^hi^ cells lie adjacent to nerve fibers, and Lyve1^hi^MHCII^lo^ cells colocalize with vasculature, protecting against the development of fibrosis. The kidney is a highly vascularized organ, necessitated by its homeostatic role of waste and acid removal from blood, but whether MoMacs in the kidney differentiate along these 2 distinct cell fate trajectories and how this might be perturbed in lupus nephritis are currently unclear.

Previous data suggest that F4/80^hi^ prenatally seeded TrMacs are the cells within the kidney that take up circulating ICs, acquiring them from endothelial cells to which they are closely opposed (17, 20). Notably, these studies used model ICs, where the antigen/antibody ratio used would generate small ICs, whereas medium and large ICs are commonly observed in lupus (21, 22). In addition, the ICs used in these studies did not contain nucleic acids or DNA (that can activate other innate receptors) (23, 24) and may therefore not truly recapitulate kidney macrophage responses in lupus nephritis. Here, using a combination of flow cytometric analysis, high-dimensional imaging, spatial transcriptomics (ST), and scRNA-Seq, we sought to delineate kidney macrophage heterogeneity, activation, signaling networks, and cell fate trajectories in a murine model of SLE, the MRL/*MpJ*-*Fas^lpr^*/J (MRL-*Lpr*) mouse, and to integrate this information with publicly available single-cell transcriptional data from human lupus nephritis to identify novel, cell type–specific therapeutic targets.

## Results

### Expanded kidney macrophage populations in lupus nephritis.

**Figure 1:**
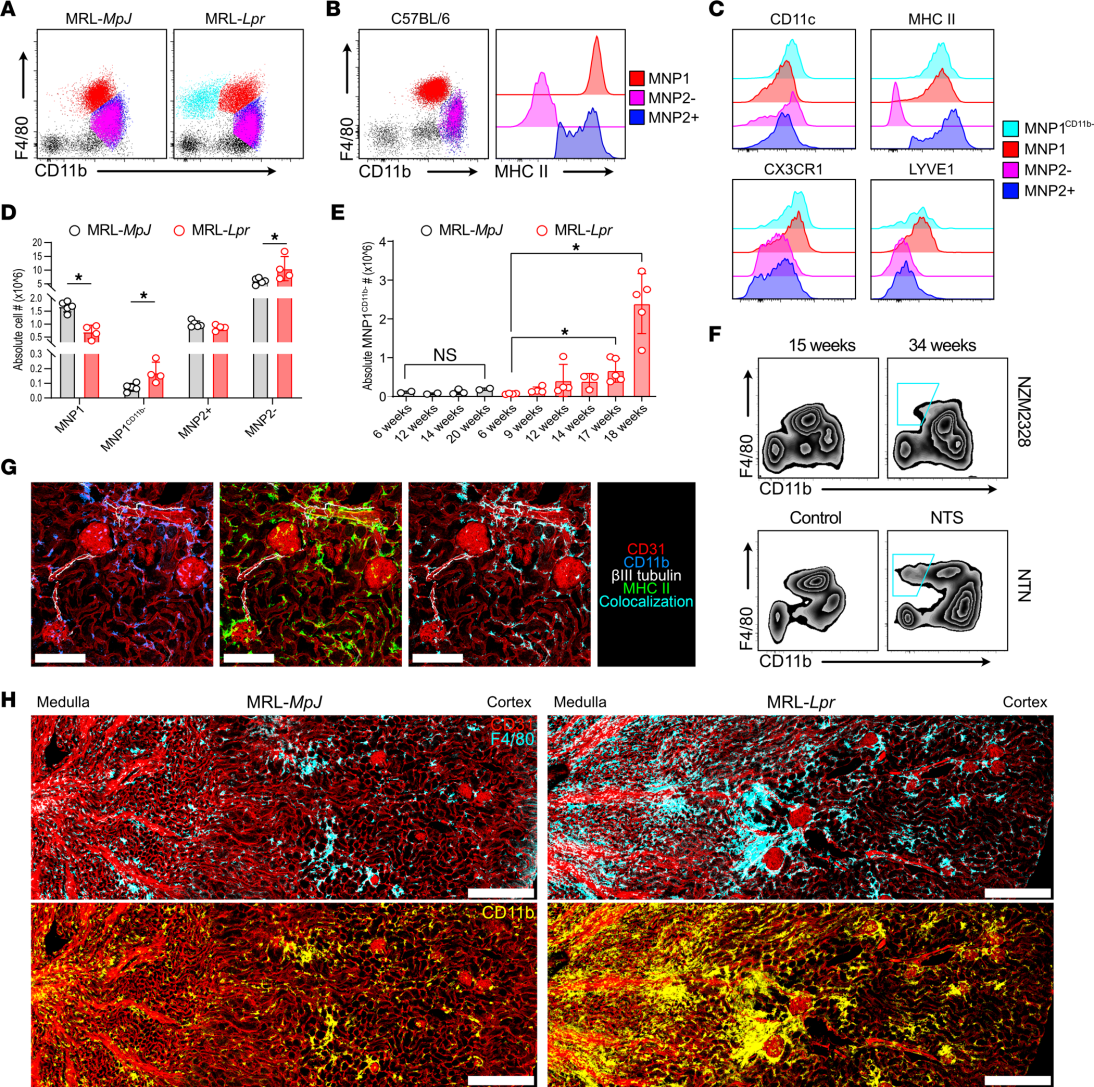
Expanded kidney macrophage populations in lupus nephritis. (**A**) Representative CD11b versus F4/80 expression by kidney MNPs in MRL-*MpJ* (left) and MRL-*Lpr* (right) mice. (**B**) Representative CD11b versus F4/80 and MHCII expression by kidney MNPs in C57BL/6 mice with cells identified as MNP1 (F4/80^hi^CD11b^int^MHCII^hi^), MNP2^+^ (F4/80^int^CD11b^hi^MHCII^+^), and MNP2^–^ (F4/80^int^CD11b^hi^MHCII^–^). (**C**) Surface expression of CD11c, MHCII, CX3CR1, and LYVE-1 by kidney MNP subsets in MRL mice. (**D**) Absolute cell numbers of kidney MNPs in 16-week-old MRL-*MpJ* and MRL-*Lpr* mice. *n* = 5–6 animals per group from 2 separate experiments. *P < 0.05, Mann-Whitney test with Benjamini, Krieger, and Yekutieli posttest. (**E**) Absolute numbers of MNP1^CD11b-^ per kidney in MRL-*MpJ* and MRL-*Lpr* mice with age. *n* = 2–5 animals per group. *P < 0.05, Mann-Whitney test. (**F**) Representative CD11b and F4/80 expression by kidney MNPs in young and old NZM2328 mice (top, *n* = 5) and following administration of nephrotoxic serum in C57BL/6 mice (bottom, *n* = 6). The cyan outline represents the MNP1^CD11b–^ population in the 34-week-old NZM2328 mouse and the mouse that received NTS. (**G**) Representative confocal microscopy (*n* = 3) on murine C57BL/6 kidney showing MNP distribution around peritubular, afferent/efferent blood vessels and glomeruli (CD31, red) as well as surrounding nerves (βIII tubulin, white) through expression of CD11b (blue) and MHCII (green). Scale bar = 150 μm. (**H**) Representative confocal microscopy (*n* = 4) showing MNP distribution in kidneys from 18-week-old MRL-*MpJ* (left) and MRL-*Lpr* (right) mice through expression of F4/80 (cyan) and CD11b (yellow) relative to blood vessels (CD31, red). Scale bar = 300 μm. int, intermediate; NTS, nephrotoxic serum; NTN, nephrotoxic nephritis.

We profiled kidney macrophages in MRL-*MpJ* controls and in diseased MRL-*Lpr* mice that harbor a mutation in *Fas*, which leads to the survival of autoreactive lymphocytes, the development of anti-nuclear/anti-DNA antibodies, and an IC-mediated glomerulonephritis, modeling lupus nephritis (25). Flow cytometric assessment indicated 2 major kidney MNP populations, an F4/80^hi^CD11b^int^ population (MNP1) and an F4/80^lo^CD11b^hi^ population (MNP2), previously reported to be yolk sac or hematopoietic stem cell/monocyte derived, respectively (11), in both MRL and C57BL/6 mice (Figure 1, A and B). In all strains examined, the MNP2 subset included an MHCII-positive and -negative population, referred to hereafter as MNP2^+^ and MNP2^–^ (Figure 1, B and C). In contrast to the previous description that 2 distinct populations of MoMacs in lungs, heart, liver, and skin demonstrate binary expression of MHCII, LYVE-1, and CX3CR1 in homeostasis (19), kidney MNP2^+^ and MNP2^–^ populations expressed similar levels of LYVE-1 and CX3CR1 (Figure 1C).

In MRL-*Lpr* mice, a distinct subset of F4/80^hi^ cells was evident, with lower expression of CD11b (designated MNP1^CD11b–^) (Figure 1, A and D). MNP1^CD11b–^ were infrequent in control kidneys, increased with age (Figure 1, D and E), and were expanded in other models of antibody-mediated nephritis (NZM2328 and nephrotoxic nephritis) (Figure 1F). In addition to this expanded MNP1^CD11b–^ population, MNP2^–^ were increased in the kidneys of MRL-*Lpr* mice (Figure 1D), suggesting an increase in both MoMac and TrMac populations in lupus nephritis.

Spatially, in control kidneys (both MRL-*MpJ* and C57BL/6), MNP subsets were asymmetrically distributed, with CD11b^hi^ MNPs enriched in the outer medulla, and present in occasional clusters in the cortex and around glomeruli, while F4/80^hi^CD11b^int/lo^ (F4/80^hi^CD11b^int/lo^) predominated in the cortex (Figure 1, G and H, and Supplemental Figure 1, A and B; supplemental material available online with this article; https://doi.org/10.1172/jci.insight.159751DS1). CD11b^hi^MHCII^+^ cells were observed adjacent to interlobular arteries, peritubular capillaries, and nerves (Figure 1G and Supplemental Figure 1A). Thus, MHCII and LYVE-1 do not appear to mark macrophages located adjacent to nerves or blood vessels, respectively, in the kidney. In MRL-*Lpr* kidneys, extensive cortical F4/80^–^CD11b^hi^ infiltrates were prominent, with some large aggregates that included F4/80^hi^CD11b^–^ cells (Figure 1H). In addition, F4/80^hi^CD11b^–^ cells prominently encased every glomerulus (Figure 1H and Supplemental Figure 1B), altogether consistent with the increase in MNP1^CD11b–^ and MNP2^–^ identified by flow cytometric assessment (Figure 1, C and D).

### Lupus nephritis–associated macrophage heterogeneity at single-cell resolution.

**Figure 2:**
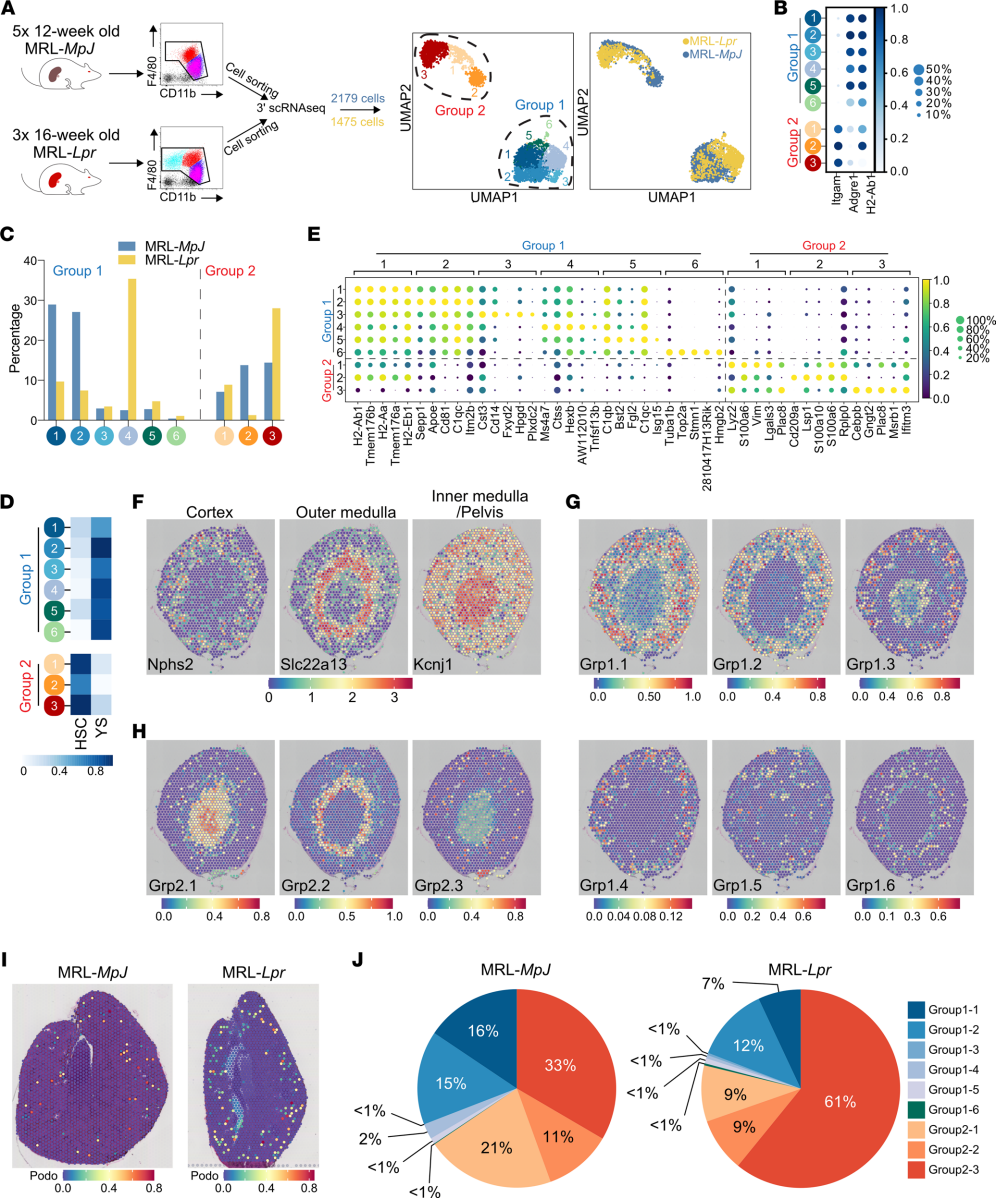
Lupus nephritis–associated macrophage heterogeneity at single-cell resolution. (**A**) Illustration of single-cell experiment setup (left panels) and uniform manifold approximation and projection (UMAP) embedding of 2,179 macrophages from MRL-*MpJ* and 1,475 macrophages from MRL-*Lpr* mice (right panels). (**B**) Mean expression dot plot of genes Itgam (CD11b), Adgre1 (F4/80), and H2-Ab1 (MHCII). (**C**) Proportion of cells found in each cluster and mouse strain. (**D**) Heatmap of mean AUCell enrichment of F4/80^hi/lo^ gene sets, corresponding to yolk sac (YS) versus hematopoietic stem cell (HSC) lineage. (**E**) Mean expression dot plot of top 5 significant marker genes for each MNP cluster. Marker genes were identified using Wilcoxon rank sum test, and *P* (adj) < 0.05 was considered statistically significant. (**F**) Spatial expression of markers used to delineate the anatomical regions in Visium Spatial Gene Expression data of C57BL/6 kidney sections (Kcnj1 — pelvis, Slc22a13 — proximal tubules, and Nphs2 — glomeruli). (**G**) Spatial transcriptomics of group 1 macrophage signatures in C57BL/6 murine kidneys with annotated scRNA-Seq data above. Each spot/voxel denotes a prediction score of 0–1 for the location of each of the macrophage subgroups. (**H**) Spatial transcriptomics (ST) of group 2 macrophage signatures in C57BL/6 murine kidneys with annotated scRNA-Seq data above. Each spot/voxel denotes a prediction score of 0–1 for the location of each of the macrophage subgroups. (**I**) ST of podocyte signature in MRL-*MpJ* (left) and MRL-*Lpr* (right) kidneys to identify glomerulus-containing spots/voxels. (**J**) Average proportion of each macrophage subset signature in spots/voxels identified in **I** in the MRL-*MpJ* (left) and MRL-*Lpr* (right) kidneys.

To more comprehensively characterize kidney MNP heterogeneity in health and to explore how these populations change in lupus nephritis, we performed droplet encapsulation scRNA-Seq (Chromium, 10x Genomics) on flow-sorted kidney macrophages isolated from MRL-*MpJ* and MRL-*Lpr* mice (6) (Figure 2A). Subclustering of the 2 major groups we described previously (group 1, MNP1, and group 2, MNP2) revealed 9 cell clusters (Figure 2A). Notably, MNP1 clusters expressed *Adgre* (F4/80) but little *Itgam* (CD11b), and vice versa, MNP2 clusters showed minimal expression of *Adgre* (F4/80) but expressed *Itgam* (CD11b) (Figure 2B), analogous to the 2 major macrophage populations identified by flow cytometry. Group 2 MNPs included a monocyte cluster (2.1), as well as MHCII-positive (MNP2.2) and -negative (MNP2.3) clusters (Figure 2, B and C), mirroring MNP2^+^ and MNP2^–^ subsets identified by flow cytometry (Figure 1) and the MoMac populations identified in other organs using scRNA-Seq (19). All MNP1 clusters showed transcriptional similarity with a reference fate-mapped, yolk sac–derived kidney macrophage signature (11) and MNP2 clusters with a fate-mapped kidney monocyte-derived macrophage signature (Figure 2D).

Differentially expressed genes in MNP1.6 included cell cycle genes (Figure 2E), indicating proliferating cells, and other canonical macrophage and monocyte markers (*Itgax* [CD11c], *Cd14*, *Ly6c*, *Ccr2*, and *Cx3cr1*) showed distinct expression patterns with little expression of DC-associated markers (Figure 2E). Each cluster contained cells from both control and diseased kidneys, although MNP1.4 (which did not express *Itgam*, Figure 2B) and MNP2.3 were markedly enriched in diseased samples (Figure 2C), consistent with our flow cytometric and confocal imaging studies, which also demonstrated an expansion of subsets of F4/80^hi^ macrophages (MNP1^CD11b–^) and CD11b^hi^MHCII^–^ macrophages (MNP2^–^) in MRL-*Lpr* kidneys (Figure 1).

We next used ST to delineate the anatomical localization of macrophage subsets in homeostasis. Podocyte (*Nphs2*), proximal tubular (*Slc22a13*, *Slc34a1*), and distal tubule/collecting duct (*Kcnj1*, *Aqp2*) gene expression delineated the anatomical regions of the kidney (Figure 2F and Supplemental Figure 2, A and B). In control kidneys, group 1 MNP subset signatures were predominantly enriched in the cortex, except for the proliferating MNP1.6 genes, which were enriched in the medulla (Figure 2G and Supplemental Figure 2C). Group 2 MNP signatures showed distinct localization patterns, with MNP2.1 (monocytes) signatures enriched in the pelvic region and MNP2.2 enriched in the outer medulla, while high MNP2.3 signatures were scattered around the cortex (Figure 2H and Supplemental Figure 2D). Using a podocyte signature (26) to specifically select glomerulus-containing voxels (Figure 2I), we examined the proportion of group 1 and 2 MNPs that localized to glomeruli and found a large increase in glomerulus-associated MNP2.3 signatures in the MRL-*Lpr* compared with the MRL-*MpJ* kidney (Figure 2J), consistent with a contribution to glomerulonephritis.

### Kidney macrophages show deranged cell state trajectories in lupus nephritis.

**Figure 3:**
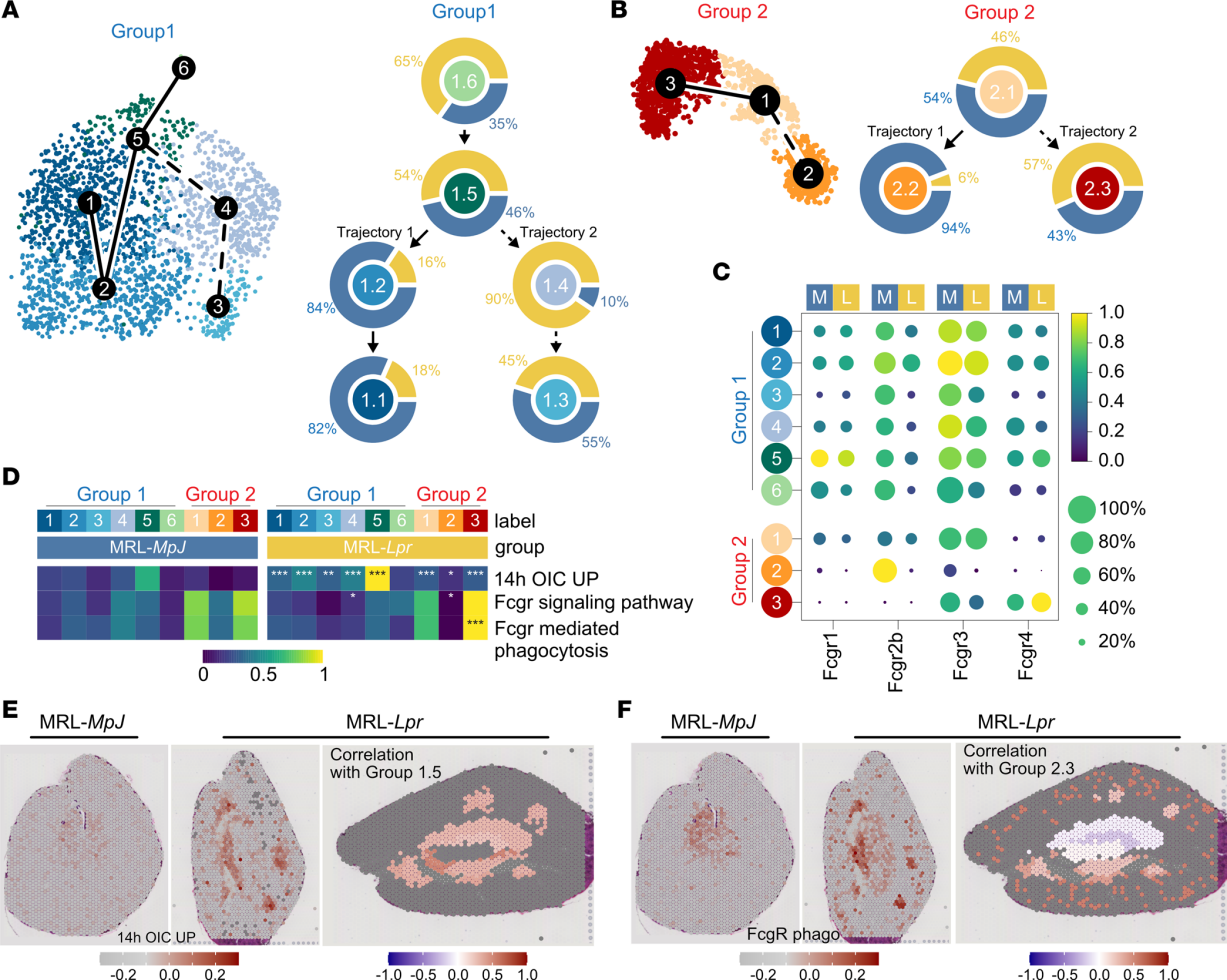
Kidney macrophages show deranged cell state trajectories in lupus nephritis. (**A**) (Left) UMAP embedding of group 1 cells with summary tracks of slingshot trajectory connecting the group 1 clusters. (Right) Proportion of cells from MRL-*MpJ* (blue) or MRL-*Lpr* (yellow) from each cluster organized according to predicted trajectory. (**B**) (Left) UMAP embedding of group 2 cells with summary tracks of slingshot trajectory connecting the group 2 clusters. (Right) Proportion of cells from MRL-*MpJ* (blue) or MRL-*Lpr* (yellow) from each cluster organized according to predicted trajectory. (**C**) Mean expression dot plot of FcγR genes split by mouse strain (M = MRL-*MpJ*; L = MRL-*Lpr*). (**D**) Heatmap of mean AUCell enrichment of immune complex (IC) stimulation/cross-linking related gene sets split by MRL-*MpJ* or MRL-*Lpr*. Statistical significance testing was performed using pairwise Wilcoxon rank sum tests and adjusted with Bonferroni posttest where *P < 0.05; **P < 0.01; ***P < 0.001. (**E**) (Left and middle panels) Spatial expression of IC stimulation/cross-linking related gene sets in Visium Spatial Gene Expression data of MRL-*MpJ* (left) and MRL-*Lpr* (right) kidney sections. (Right panel) Positive correlation values of expression of gene set with group 1.5 prediction scores across k-nearest neighborhoods of spots are colored from white to red. Gray spots indicate no available scores or negative correlation. (**F**) (Left and middle panels) Spatial expression of FcγR-mediated phagocytosis-related gene sets in Visium Spatial Gene Expression data of MRL-*MpJ* (left) and MRL-*Lpr* (right) kidney sections. (Right panel) Positive correlation values of expression of gene set with group 2.3 prediction scores across k-nearest neighborhoods of spots are colored from white to red. Gray spots indicate no available scores or negative correlation. OIC, opsonized immune complex; UP, upregulated.

The developmental trajectories of the F4/80^hi^ group 1 clusters showed a progression from the proliferating cluster MNP1.6, through to MNP1.5 and then a binary divergence to either MNP1.2 and MNP1.1 or to MNP1.4 and MNP1.3 (Figure 3A), with a marked skewing toward the latter trajectory in MRL-*Lpr* kidneys (Figure 3A and Supplemental Figure 3A). Transcription factor (TF) regulon activity analysis at the divergence of pseudotime cell fate showed *Maf*-, *Runx1*-, *Nfatc2*-, *Irf4*-, and *Irf7*-regulated genes were highly expressed by cells transitioning from MNP1.5 to MNP1.2 (Supplemental Figure 3B), while *Cebpg*, *Junb*, *Irf8*, and *Stat1* were expressed in cells transitioning from MNP1.5 to MNP1.4 (Supplemental Figure 3B). Of note, *Maf* (also known as cMaf), together with MafB, desensitize macrophages to proliferative stimuli, such as M-CSF, by inhibiting the expression of genes such as *Myc* (27), promoting a quiescent tissue macrophage phenotype. Furthermore, *Maf* and *Nfact2c* have been shown to be upregulated in renal macrophages as they differentiate from progenitors following seeding in the embryo, with the latter a kidney-specific TF (18). In contrast, IRF8 and STAT1 mediate inflammatory M1 polarization in macrophages (28), activated by IFN-γ stimulation (29).

Chakarov et al. used scRNA-Seq to investigate MoMacs in lungs, skin, and heart and showed that MHCII^+^ and MHCII^–^ cells represented 2 distinct cell developmental fates rather than cells at opposite ends of a cell differentiation spectrum (19). Similarly, in the kidneys, we found that MNP2.2 and MNP2.3 developed from monocytes (MNP2.1) along 2 distinct trajectories, with the 2.1 to 2.3 trajectory favored in lupus nephritis (Figure 3B and Supplemental Figure 3C). TF regulon activity showed increased expression of Irf4- and Stat6-controlled genes as cells moved from MNP2.1 (monocytes) to MHCII^+^ MNP2.2 (Supplemental Figure 3D), TFs known to polarize toward an antiinflammatory M2 macrophage phenotype (28). In contrast, Stat3-regulated genes increased in cells progressing toward MNP2.3 (MHCII^–^) cluster (Supplemental Figure 3D), with STAT3 activated by a number of cytokines, including IL-6 (30).

### MoMacs are IC responders in lupus nephritis.

Interestingly, NF-κB1–regulated genes were also increased in MoMacs as they differentiated from monocytes, a TF activated by FcγR cross-linking in phagocytes (31). (Supplemental Figure 3D). Indeed, *Fcgr4* (an activating FcγR) expression was substantially higher in MNP2.3 in MRL-*Lpr* compared with MRL-*MpJ* kidneys (Figure 3C), with a marked reduction in the inhibitory *Fcgr2b* in all TrMac and MoMa subsets (particularly MNP2.2) in MRL-*Lpr* kidneys, with the overall effect of increasing macrophage activation upon IgG IC encounter. Consistent with this, we observed a significant enrichment of a macrophage IgG IC stimulation gene signature across all group 1 and group 2 MNP clusters in MRL-*Lpr* mice compared with MRL-*MpJ* mice, particularly in MNP1.5 (Figure 3D). Furthermore, Gene Ontology– and Kyoto Encyclopedia of Genes and Genomes–curated gene sets for FcγR signaling and FcγR-mediated phagocytosis were strongly enriched in MNP2.3 in MRL-*Lpr* kidneys (Figure 3D). ST analysis supported a marked increase in IC-induced gene signatures in MRL-*Lpr* kidneys that colocalized with the MNP1.5 gene signature (Figure 3E), as well as an increase in expression of the FcγR-mediated phagocytosis gene set in the cortex that colocalized MNP2.3 gene signature (Figure 3F). Together these data support the conclusion that disease-associated subsets of both TrMacs and MoMacs are responding to IgG IC stimulation via FcγR cross-linking in lupus nephritis.

**Figure 4:**
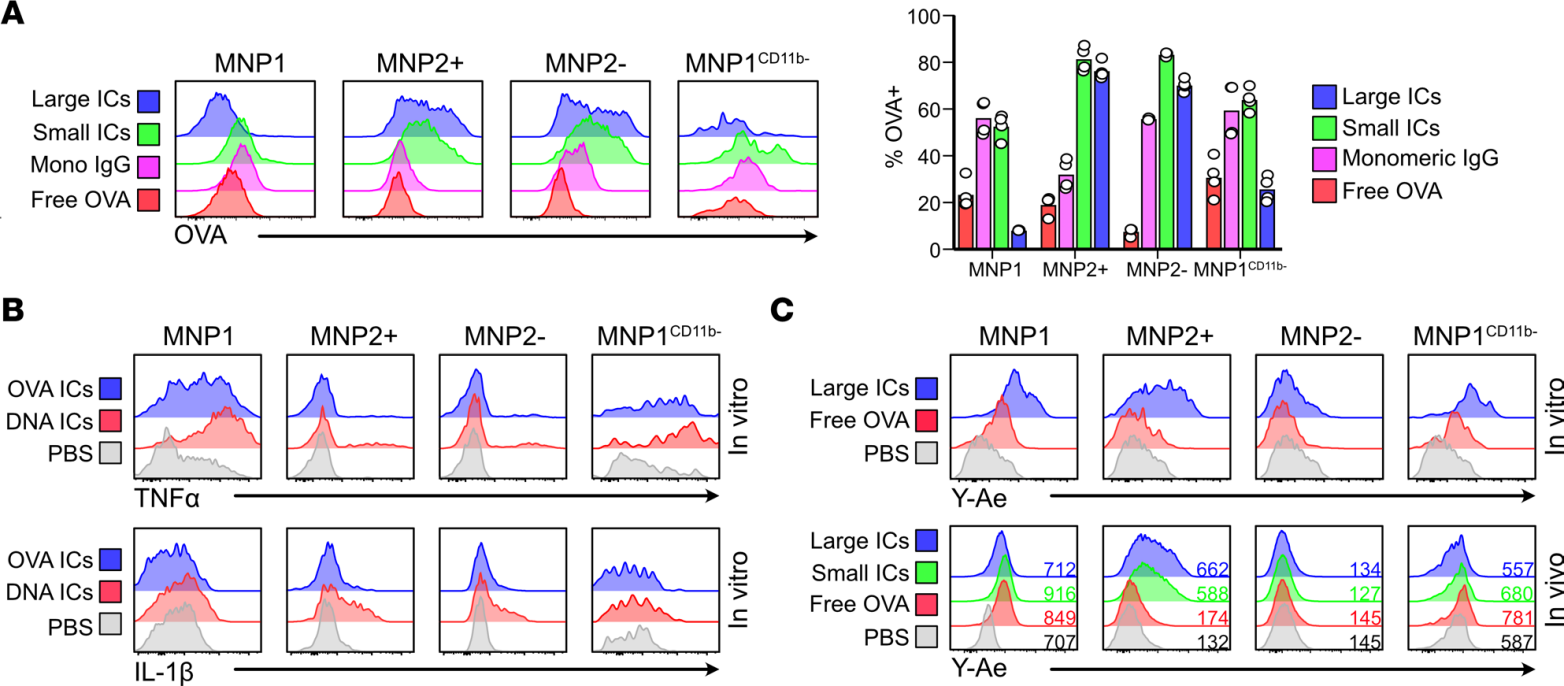
Kidney macrophages produce cytokines and present antigen upon IC uptake. (**A**) (Left) Uptake of free and immune complexed AF647-OVA by kidney MNPs in vivo 2 hours following intravenous injection in C57BL/6 mice. 5:1 (large), 1:1 (small), and 1:5 (monomeric) molar ratios of rabbit IgGs/OVA were used. (Right) Uptake of free and immune complexed AF647-OVA by kidney MNPs in vivo 2 hours following intravenous injection in C57BL/6 mice. *n* = 2–4 mice per group from 2 separate experiments. (**B**) Representative expression (*n* = 3) of TNF-α (top) and IL-1β (bottom) by each MNP subset after 2 hours of stimulation in vitro with the appropriate condition. (**C**) Antigen presentation by kidney MNPs in vitro (top, *n* = 3) 2 hours following stimulation with free or immune complexed Eα-AF647-OVA or in vivo (bottom, *n* = 4–6 from 2 separate experiments) 4 hours following intravenous injection of free or immune complexed Eα-AF647-OVA in C57BL/6 mice. The number indicates the average gMFI for this channel. AF647, Alexa Fluor 647; gMFI, geometric mean fluorescence intensity.

Given that our scRNA-Seq and ST analysis showing MoMacs as IC responders was in contrast to previous reports implicating prenatally seeded TrMac as the principal cell type capable of internalizing ICs (17, 20), we sought to directly assess the capacity of kidney macrophages to internalize circulating ovalbumin (OVA) ICs, generated using varying ratios of rabbit anti-OVA IgG. Following in vivo challenge by intravenous administration of ICs, F4/80^hi^ TrMacs internalized free OVA and small ICs (IgG/OVA ratio of 1:1 and 1:10) but demonstrated little uptake of larger ICs (IgG/OVA ratio of 5:1) (Figure 4A). In contrast, MoMac subsets readily internalized large IgG ICs from the circulation (Figure 4A). To confirm that this observation was not limited to rabbit IgG ICs, we generated larger ICs using mouse anti-OVA IgG and similarly showed that MoMac subsets readily internalized circulating ICs with no significant uptake by F4/80^hi^ TrMacs in vivo (Supplemental Figure 3E). In vitro, FcγR cross-linking by OVA ICs and (to an even greater extent) anti-dsDNA-DNA ICs induced the production of TNF-α, and IL-1β, by both TrMacs and MoMacs (Figure 4B). In the scRNA-Seq data, group 1 and group 2 MNPs expressed AIM2, which may contribute to the augmented responses to anti-dsDNA ICs (Supplemental Figure 3F). Both TrMacs and MoMacs were able to present immune complexed Y-Ae (32) in vitro, but in vivo TrMacs did not present IV immune complexed antigen. Instead, MoMacs were the major antigen presenters (Figure 4C). Together, these data implicate MoMacs as IC-phagocytosing and -responding populations in the kidney in vivo.

### TrMacs orchestrate monocyte recruitment in lupus nephritis.

**Figure 5:**
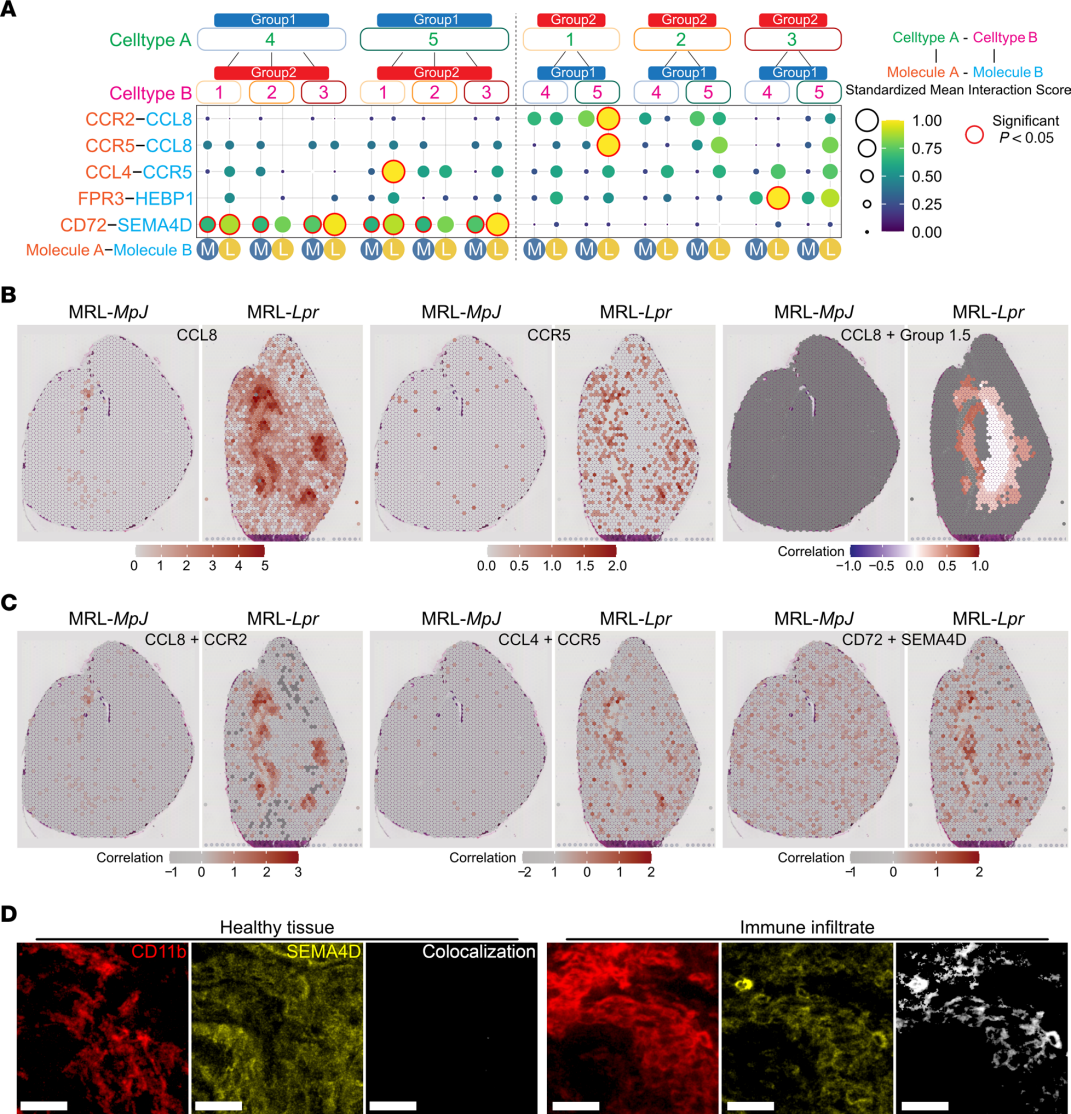
TrMacs orchestrate monocyte recruitment in lupus nephritis. (**A**) CellPhoneDB receptor-ligand interaction analysis between group 1 (group 1.4 and group 1.5) and group 2 (group 2.1, group 2.2, and group 2.3) clusters split by mouse strain (M = MRL-*MpJ*; L = MRL-*Lpr*). Significant interactions (*P* < 0.05) are highlighted in red. (**B**) Spatial expression of CCL8 and CCR5 in Visium Spatial Gene Expression data of MRL-*MpJ* (left) and MRL-*Lpr* (right) kidney sections. (Left and middle) Expression of molecules per spot colored from increasing gradient from white to red, corresponding to increasing expression value. (Right) Positive correlation values of expression of molecules with Group 1.5 prediction scores across k-nearest neighborhoods of spots are colored from white to red. Gray spots indicate no available scores or negative correlation. (**C**) Positive correlation values of expression of molecules (CCL8 and CCR2, left; CCL4 and CCR5, middle; CD72 and SEMA4D, right) in Visium Spatial Gene Expression data of MRL-*MpJ* (left) and MRL-*Lpr* (right) kidney sections. Positive correlation values of expression of molecules are colored from white to red. Gray spots indicate no available scores or negative correlation. (**D**) Representative confocal microscopy (*n* = 3) showing SEMA4D expression (yellow) in healthy tissue (left) and a large immune infiltrate (right) in the cortex (left, CD11b — red) in the kidneys from 18-week-old MRL-*Lpr* mice. Colocalization of CD11b with SEMA4D shown in white. Scale bar = 25 μm.

We next considered the role of TrMacs in lupus pathogenesis. Analysis of cell-cell interactions between mouse TrMac and MoMac subsets based on receptor-ligand expression (33) revealed the potential for MNP1.5 to recruit MNP2.1 via the expression of *Ccl4* and *Ccl8* in MRL-*Lpr* but not MRL-*MpJ* kidneys (Figure 5A). ST analysis confirmed the significance of such interactions in vivo as *Ccl8* and *Ccr5* were shown to be coexpressed in inflamed regions of MRL-*Lpr* kidneys, with *Ccl8* expression colocalizing with MNP1.5 gene signatures in these regions (Figure 5B). Similarly, *Ccl8* and *Ccr2* or *Ccl4* and *Ccr5* were also coexpressed in MRL-*Lpr* kidneys, with colocalized expression undetectable in MRL-*MpJ* kidneys (Figure 5C).

In MRL-*Lpr* kidneys, MNP1.4 also expressed *Hebp1* (Figure 5A), a molecule with chemoattractant activity via *Fpr3* (34). *Cd72*-*Sema4d*–mediated interactions were also predicted to reinforce physical associations between lupus-associated TrMac subsets (MNP1.4 and MNP1.5) and MoMac subsets in the context of nephritis (Figure 5A), which was further supported by the expression of *Sema4d* in large CD11b^hi^ MNP2 infiltrates (Figure 5D) as well as the coexpression of these molecules in MRL-*Lpr* kidneys (Figure 5C). Notably, *Sema4d* (CD100) has previously been shown to promote macrophage accumulation in glomeruli in nephrotoxic nephritis (35), and CD72 ligation by CD100 can directly activate MNPs (36).

**Figure 6:**
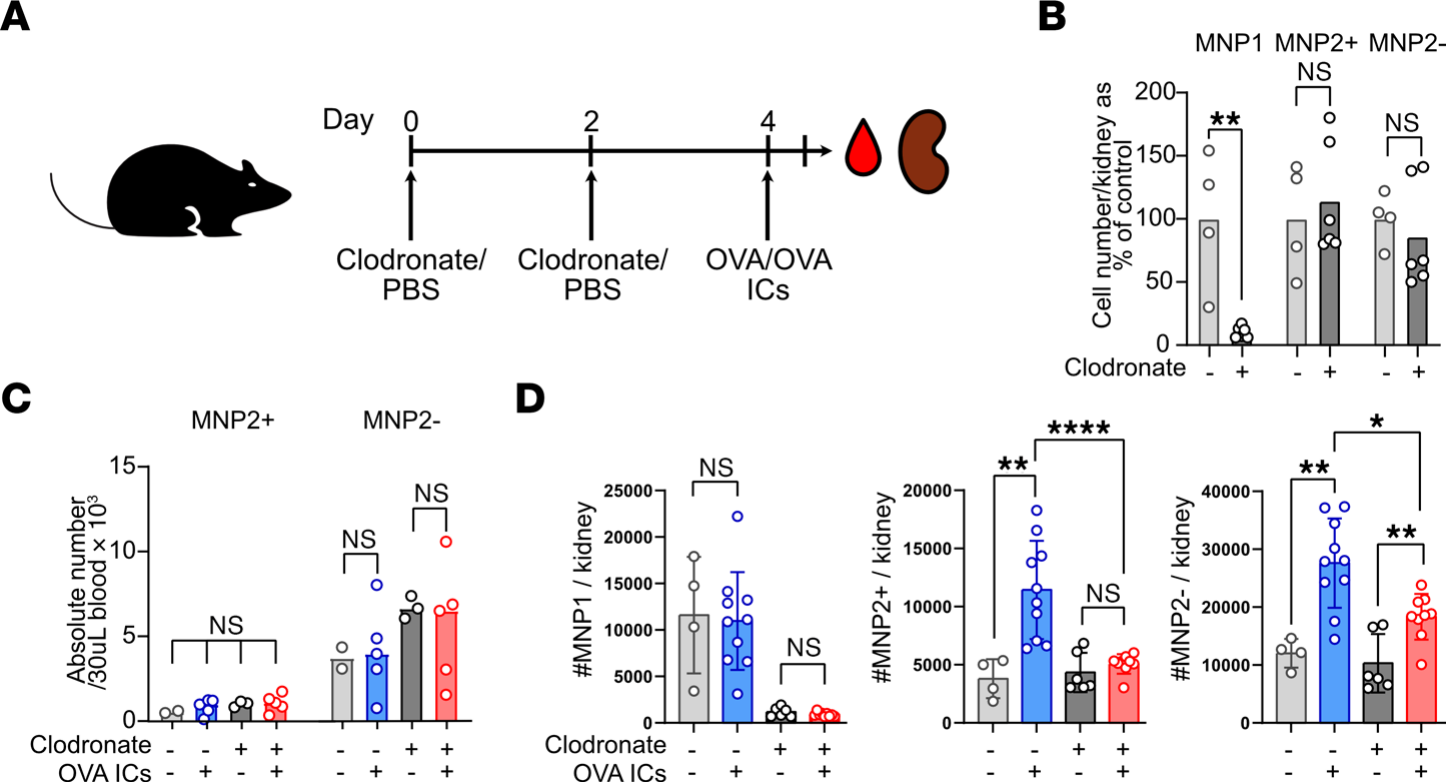
TrMacs orchestrate IC-dependent monocyte recruitment. (**A**) Illustration of the experimental setup for in vivo experiment. (**B**) Numbers of MNP1 and MNP2 subsets in the kidneys following 2 injections of liposome clodronate shown as percentage of cells in the control (PBS injected) kidneys. *n* = 4–6 per group. **P < 0.01, Mann-Whitney test with Benjamini, Krieger, and Yekutieli posttest applied. (**C**) Absolute number of CD11b^hi^MHCII^+^ (MNP2^+^) and MHCII– (MNP2^–^) cells in 30 μL of blood 4 hours following injection of free or complexed OVA in mice that had received clodronate or PBS as described in Figure 3E. *n* = 2–5 per group. (**D**) Absolute numbers for each MNP subset 4 hours following i.v. injection of free or immune complexed OVA (right) for each experimental condition in the kidneys. *n* = 4–10 per group. *P < 0.05, **P < 0.01, ****P < 0.0001, Mann-Whitney test with Benjamini, Krieger, and Yekutieli posttest applied.

To validate the importance of MNP1-MNP2 interactions in vivo following challenge with ICs, we treated mice with liposomal clodronate, which specifically depleted kidney TrMacs but had no significant effect on kidney or blood MNP2 numbers (Figure 6, A–D). Challenging mice with large ICs intravenously resulted in a significant increase in kidney MoMacs, which was substantially abrogated by prior depletion of TrMacs with liposomal clodronate (Figure 6D), demonstrating the functional importance of TrMac chemokine production in orchestrating monocyte recruitment in the context of IC stimulation.

### TrMacs produce B cell cytokines.

**Figure 7:**
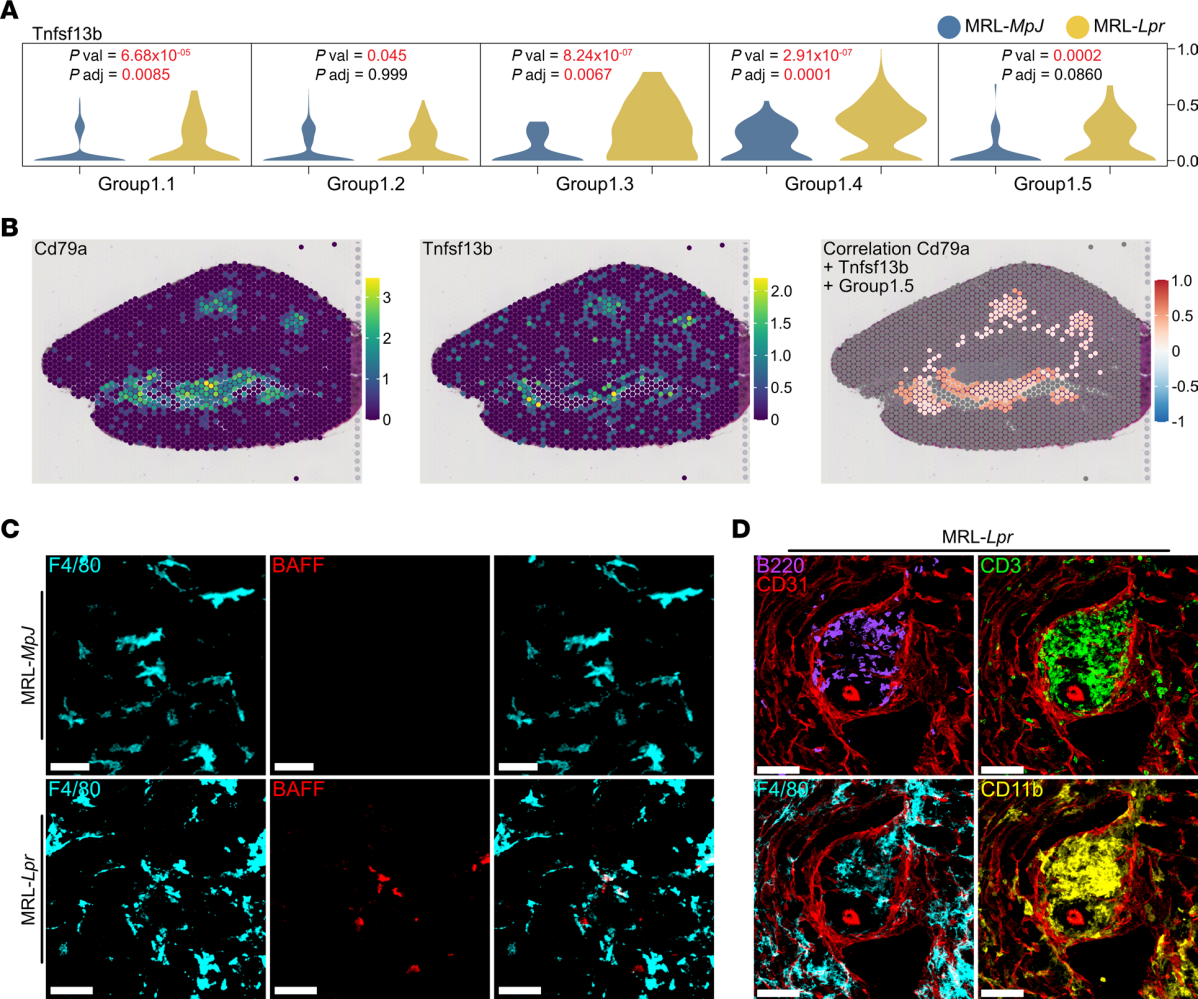
TrMacs produce B cell cytokine. (**A**) Violin plot of Tnfsf13b (BAFF) in group 1 clusters. (**B**) Spatial expression of Cd79a and Tnfsf13b in Visium Spatial Gene Expression data of MRL-*Lpr* kidney section. (Left and middle) Expression of molecules per spot are colored from increasing gradient from purple to green to yellow, corresponding to increasing expression value. (Right) Positive correlation values of expression of molecules with group 1.5 prediction scores across k-nearest neighborhoods of spots are colored from white to red. Gray spots indicate no available scores or negative correlation. (**C**) Representative confocal microscopy (*n* = 3 per group) showing BAFF expression (red) in macrophages (F4/80, cyan) in kidneys from 18-week-old MRL-*MpJ* (top) and MRL-*Lpr* (bottom) mice. Scale bar = 30 μm. (**D**) Representative confocal microscopy (*n* = 3) showing immune infiltrates containing B (B220, purple) and T (CD3, green) cells alongside F4/80^+^ (cyan) and CD11b^+^ (yellow) MNPs around blood vessels (CD31, red) in kidneys from MRL-*Lpr* mice. Scale bar = 70 μm.

In addition to inflammatory cell infiltrates, tertiary lymphoid follicles with B/plasma cell aggregates capable of local autoantibody production have been described in murine models of lupus nephritis (8, 9). We therefore asked whether kidney MNPs expressed B/plasma cell survival factors that might support such activity. We found increased expression of *Tnfsf13b*, encoding the B/plasma cell survival factor BAFF, in lupus-enriched TrMac subsets (MNP1.3, 1.4, and 1.5) in MRL-*Lpr* kidneys compared with MRL-*MpJ*, with little *Tnfsf13b* expression in MoMac subsets (Figure 7A and Supplemental Figure 4A). In the ST data, B cell clusters (evidenced through expression of *Cd79a*), were found in areas with high *Tnfsf13b* expression and specifically colocalized with MNP1.5 gene signatures (Figure 7B). BAFF expression was also detected at the protein level in F4/80^hi^ MNPs only in kidneys from MRL-*Lpr* mice (Figure 7C), with tertiary lymphoid-like structures formed of B cells, T cells, and MNPs evident in inflamed kidneys (Figure 7D).

### Human kidney MNPs show perturbations in FcyR expression, activation, and interactions in lupus nephritis.

**Figure 8:**
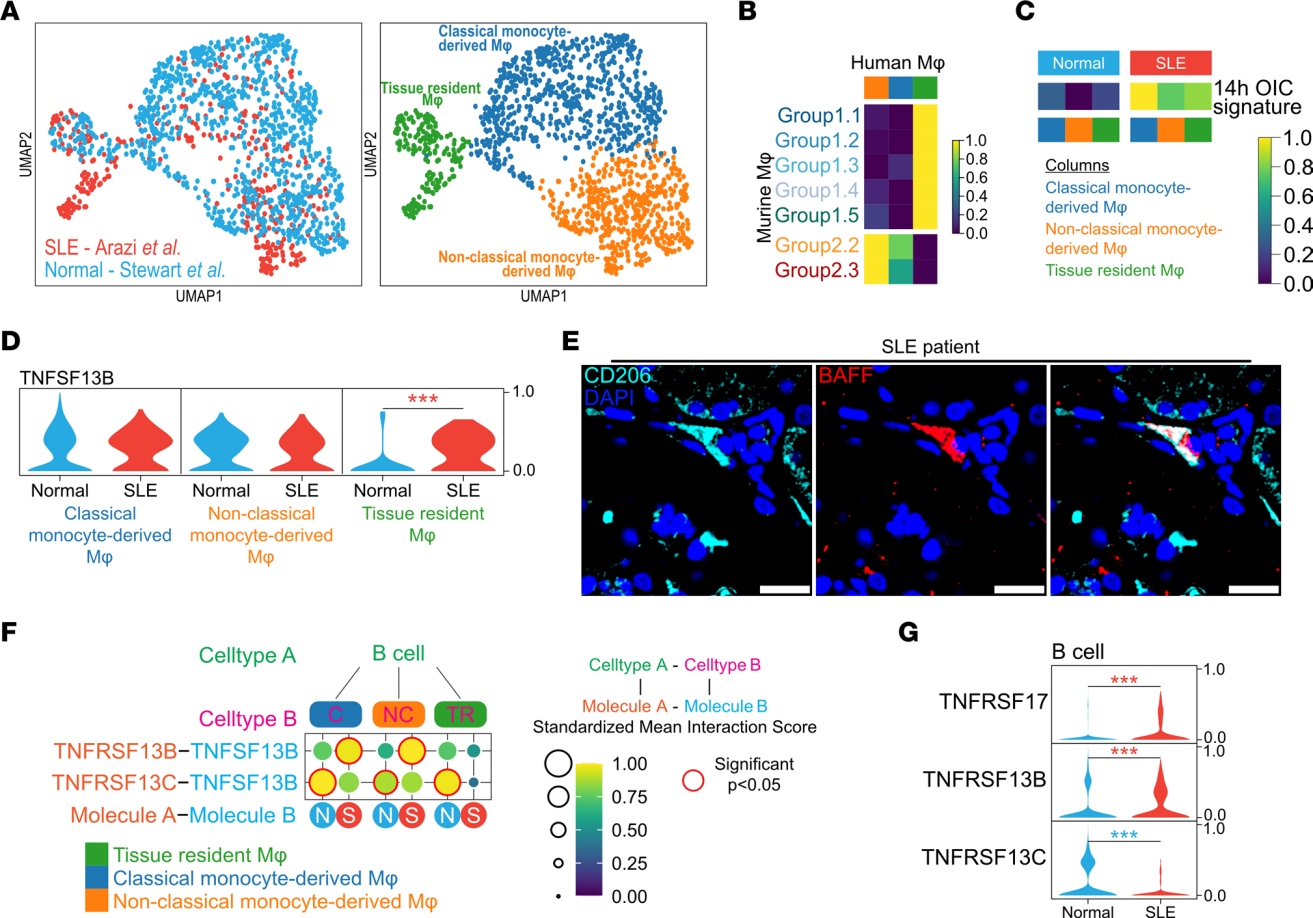
Human kidney MNPs show perturbations in FcγR expression, activation, and interactions in lupus nephritis. (**A**) Integrated UMAP embedding of macrophages from publicly available data sets of human kidneys (normal, ref. 37, and SLE, ref. 38). (**B**) Heatmap of mean AUCell enrichment of mouse group 1 and group 2 signatures in human macrophage clusters. (**C**) Heatmap of mean AUCell enrichment of OIC cross-linking signature split by normal or SLE. (**D**) Violin plot of Tnfsf13b in human macrophage clusters. Expression value is scaled from 0 to 1 across cell clusters. Significance was calculated using Wilcoxon rank sum test with BH posttest applied where ***P < 0.001. Color of the *P* value indicates which group has a higher value (red = SLE, blue = normal). (**E**) Microscopy showing BAFF expression (red) in peri-glomerular macrophages (CD206, cyan) in kidneys from SLE patients. Scale bar = 20 μm. (**F**) CellPhoneDB receptor-ligand interaction analysis between human B cells and human macrophage clusters (NC — nonclassical monocyte-derived macrophages; C – classical monocyte-derived macrophages; TR — tissue resident macrophages) split by normal (N) or SLE (S). Significant interactions (*P* < 0.05) are highlighted in red. (**G**) Violin plot of BAFF receptor molecules in human B cells. Expression value is scaled from 0 to 1. Significance was calculated using Wilcoxon rank sum test with BH posttest applied where ***P < 0.001. Color of the *P* value indicates which group has a higher value (red = SLE, blue = normal).

To validate these findings in human kidney macrophages in SLE, we integrated our data set of single-cell transcriptomes from healthy human kidney macrophages (37) with those isolated from human kidneys with lupus nephritis (38). Macrophages formed 3 major clusters with expression profiles consistent with their identity as classical monocyte-derived macrophages, nonclassical monocyte-derived macrophages, and tissue-resident macrophages, each of which contained cells from both healthy and lupus nephritis kidneys (Figure 8A). The tissue-resident cluster was enriched for YS-derived mouse kidney macrophage gene signature and the monocyte-derived clusters for HSC/monocyte-derived mouse kidney macrophage gene signature (Supplemental Figure 4B). Comparing mouse kidney macrophage transcriptomes to human macrophages, group 1 MNP signatures were enriched in the human TrMac cluster, and group 2 MNP signatures were enriched in the human MoMac clusters (Figure 8B). Macrophage IC stimulation signature genes were increased in all 3 human macrophage subsets in lupus nephritis compared with healthy kidneys, particularly in classical MoMacs (Figure 8C). Furthermore, the MRL-*Lpr*–enriched murine MNP2.3 signature was increased in human MoMacs in lupus nephritis, while the MRL-*MpJ*–enriched MNP2.2 signature was decreased (Supplemental Figure 4C).

Interestingly, as observed in murine MRL-*Lpr* MNP1 clusters, *TNFSF13B* (encoding BAFF) expression was increased in TrMacs in human lupus nephritis compared with control kidneys (Figure 8D), and macrophage BAFF expression was validated in biopsies obtained from patients with lupus nephritis (Figure 8E). Receptor-ligand expression analysis integrating kidney B cell and macrophage single-cell data supported BAFF as a major mediator of macrophage–B cell interactions but revealed differing BAFF receptor (BAFF-R) expression in lupus nephritis, with *TNFSR13B* (encoding transmembrane activator and cyclophilin ligand interactor, TACI) prominent in mediating interactions in lupus nephritis but *TNFSR13C* (encoding BAFF-R) dominant in control kidneys (Figure 8, F and G).

MoMacs in lupus nephritis biopsies showed increased expression of *CCR5* and *FRP3* in lupus nephritis (Supplemental Figure 4D), mirroring our findings in MRL-*Lpr* kidneys, and there was also increased expression of *SEMA4D* in both MoMac and TrMac subsets (Supplemental Figure 4D). All the top 10 MNP1.4 TF regulons were increased in human TrMacs in lupus nephritis, including *STAT1* (Supplemental Figure 5A), and many of the top 10 MNP2.3 TF regulons were increased in MoMacs (Supplemental Figure 5B). This included *IKZF1* (encoding IKAROS), a genetic susceptibility locus for human SLE and lupus nephritis (39, 40).

Overall, these data indicate distinct roles for TrMacs and MoMacs in lupus nephritis and show similar disruption of kidney macrophage signaling networks in mice and humans, with TrMacs promoting monocyte recruitment and providing a tissue niche for B cells (Supplemental Figure 5C).

## Discussion

Our work has several surprising findings. A previous study applying scRNA-Seq to tissue macrophages found 2 distinct populations of MoMacs in lungs, heart, liver, and skin that were MHCII^hi^LYVE-1^lo^, or vice versa, that developed along 2 distinct trajectories and occupied distinct anatomical niches adjacent to nerves and blood vessels, respectively (19). The authors suggested this was a universal phenomenon across organs, but though we similarly saw binary fate trajectories in the kidney, these 2 populations expressed similar levels of LYVE-1 and CX3CR1 and did not differentially colocalize with vasculature or nerves. In lupus nephritis, macrophage fate in these MoMacs was skewed toward the development of the MHCII^–^ subset, which showed increased *Fcgr4* expression and transcriptional evidence of high NF-κB activity and FcγR-mediated activation. This was unexpected, given previous reports suggesting that F4/80^hi^ prenatally seeded TrMacs represent the main population capable of phagocytosing circulating IgG ICs (17, 20), but when we directly challenged mice with large ICs intravenously, we showed that CD11b^hi^ MoMac subsets had substantial capacity to internalize ICs in vivo. The likely explanation for these differing observations relates to the size of IgG ICs, with previous studies utilizing small or monomeric IgG-opsonized antigens, while we profiled uptake of a range of sizes of ICs, including larger ICs that more closely resembled those found in human lupus (21, 22).

Although F4/80^hi^ TrMac subsets showed minimal IC uptake, our data indicate an important role in orchestrating tissue inflammation in lupus nephritis. Disease-enriched TrMac subsets expressed chemokines capable of orchestrating the recruitment of monocytes, validated using ST data sets, and shown to be functionally important by the demonstration that TrMacs attenuated monocyte recruitment to the kidneys after intravenous IgG IC challenge. TrMacs in mouse and human lupus nephritis also showed increased expression of *TNFSF13B*, encoding BAFF, a cytokine that enhances B cell survival and proliferation (41) and contributes to the plasma cell niche (42). BAFF mediates its effects via 3 receptors, TNFSFR13C (BAFF-R), TNFSFR17 (BCMA), and TNFSFR13B (TACI). Selective BAFF blockade prevents the development of lupus nephritis in mice (43), and the humanized anti-BAFF IgG1 antibody belimumab is licensed for use in patients with SLE (44), with potential efficacy in lupus nephritis (45). Our analysis specifically identified TrMacs as a source of kidney BAFF in mice and humans and implicated TACI in intra-renal B cell responses to BAFF in lupus nephritis but not in homeostasis, highlighting a disease-specific therapeutic target. Both TrMacs and MoMacs showed increased expression of *SEMA4D* (CD100) in lupus nephritis. Its ligand CD72 is highly expressed by B cells (46), identifying an additional axis of macrophage–B cell crosstalk that may also represent a novel therapeutic target.

In summary, our study presents a detailed analysis of the single-cell transcriptional profiles of kidney macrophages in lupus nephritis, integrating murine and human data, and providing spatial validation using ST. We define how cell fate trajectories of both TrMacs and MoMacs become deranged in disease and identify key TFs that may control cell progression toward a more proinflammatory transcriptional program. We find distinct roles for TrMacs and MoMacs, with the former showing limited IC uptake, but playing a major role in orchestrating the recruitment and maintenance of inflammation-associated cells, and the latter being able to internalize and present IC-associated antigen. Overall, this work provides a single-cell and spatial transcriptional atlas for macrophages in lupus nephritis and identifies potentially novel cell subset–specific therapeutic targets.

## Methods

### Mice

Wild-type C57BL/6 mice were obtained from The Jackson Laboratory. Transgenic mice expressing Venus EYFP under the control of the CD11c promoter were a gift from M Nussenzweig (Rockefeller University, New York, New York, USA). NZM2328 mice were a gift from MJ Kaplan (National Institute of Arthritis and Musculoskeletal and Skin Diseases, National Institutes of Health, NIAMS/NIH; Bethesda, Maryland, USA). MRL-*MpJ* (no. 00486) and MRL-*Lpr* (no. 00485) mice were obtained from The Jackson Laboratory. In all experiments, both male and female mice were used. For all in vivo experiments, 8- to 16-week-old mice were used. In the United Kingdom, mice were maintained in specific pathogen–free conditions at a Home Office–approved facility.

### Murine kidney processing

Following terminal procedure, mouse kidneys were minced finely and digested in RPMI (MilliporeSigma) containing 0.1 mg/mL DNase I (Merck), 0.0325 mg/mL liberase (Merck), and 10 mM HEPES for 25 minutes at room temperature. Organs were then mechanically dissociated through a 70 μm cell strainer (Falcon, Corning), then washed in PBS, and red blood cell lysis was performed. Single-cell suspension was enriched in hematopoietic cells by Percoll gradient centrifugation (2,000 rpm in 44% Percoll in RPMI 10% FBS with centrifuge break on 0 for 20 minutes), pellet was washed with cold PBS 2% FBS, and cells were then used for further analysis.

### Flow cytometry

Single-cell suspensions were blocked for 30 minutes with 50 μL normal mouse serum in PBS 2% FBS on ice, then stained with the appropriate fluorescently labeled antibodies (see antibody table) and live/dead staining for 30 minutes on ice. When using biotinylated antibodies, cells were then washed and stained with fluorescently labeled streptavidin for 20 minutes on ice. After washing, cells were analyzed on an LSRFortessa (BD Biosciences). FCS files were analyzed using FlowJo v9.9.6. Antibodies used in this study are in Supplemental Table 1.

### Cell sorting

Stained samples were sorted on a FACSAria III (BD Biosciences) following the appropriate gating strategy. Cells were sorted under the 4-way purity setting into chilled FBS and kept on ice until ready to use.

### Generation of OVA ICs

ICs were prepared in vitro by incubating AF647-OVA (1 mg/mL in PBS) or DQ-OVA (1 mg/mL in PBS) with rabbit polyclonal anti-OVA antiserum (3.7 mg/mL) at a 1:10, 1:5, 1:1, 5:1, or 10:1 molar ratio for 60 minutes in a 37°C water bath. Large ICs (1:5 and 1:10 molar ratios) were washed twice (10,000 rpm for 2 minutes with discarding of the supernatant) prior to being injected to remove excess OVA or excess IgG.

### Generation of DNA ICs

ICs were prepared in vitro by incubation with Ultrapure salmon sperm DNA (Thermo Fisher Scientific) and sonicated into fragments ranging from 150 to 300 bp with anti-dsDNA antibodies engineered to have a human IgG1 backbone for 60 minutes in a 37°C water bath. The anti-dsDNA antibodies were provided by the autoimmunity, transplantation, and inflammation branch at Novartis, Switzerland.

### In vitro uptake by kidney MNPs

Single-cell suspension was prepared as described. Cells were then resuspended in prewarmed RPMI 10% FBS and plated in a 24-well plate, 3 × 10^6^ cells per well in 300 μL medium. Cells were given opsonized/free OVA in 10 μL PBS and incubated in the dark at 37°C for the appropriate time. After incubation, cells were washed with cold PBS 2% FBS and stained for flow cytometry analysis.

### In vivo uptake by kidney MNPs

All compounds were administered intravenously via tail vein injection in a final volume of 100 μL. ICs of different sizes were prepared as described so that each mouse received 8 μg worth of AF647-OVA. The amount of anti-OVA serum used to form ICs was calculated to fit the desired molar ratios. Two hours after injection, mice were culled via cervical dislocation. Both kidneys and blood from the renal vein were harvested for further processing.

### Antigen presentation

Eα peptide (sequence CGGGASFEAQGALANIAVDKA) was conjugated to AF647-OVA (termed Eα-OVA) on custom order (ALMAC). Eα-OVA was mixed at a 1:1 ratio with AF647-OVA for all antigen presentation experiments. Presentation was assessed using the Y-ae antibody clone for flow cytometry.

### Confocal microscopy of human samples

Biopsies were processed and embedded in paraffin. Before staining, 10 μm sections were heated 30 minutes at 65°C and then rehydrated as follows: xylene — 10 minutes, 2 changes; 100% ethanol — 10 minutes, 2 changes; 95% ethanol — 5 minutes; 70% ethanol — 5 minutes; 50% ethanol — 5 minutes, washed with deionized water, and rehydrated with PBS 1× — 10 minutes. Antigen retrieval was performed using citrate buffer 100× pH 6.0 (Abcam ab93678) 99°C — 40 minutes. Sections were incubated with primary antibodies for 2 hours at room temperature (RT) and washed 3 times in PBS. Finally, sections were incubated with the appropriate fluorochrome-labeled secondary antibodies for 1 hour at RT, washed in PBS, and mounted in Fluoromount-G with DAPI (Invitrogen). Images were acquired using a TCS SP8 (Leica Microsystems) confocal microscope. Raw imaging data were processed using Imaris (Bitplane). Antibodies used in this study are in Supplemental Table 1.

### Confocal microscopy of murine samples

Samples were fixed in 1% paraformaldehyde (Electron Microscopy Services)/l-lysine/sodium periodate (both MilliporeSigma) buffer for 24 hours or in AntigenFix for 1 hour at 4°C followed by 8 hours in 30% sucrose in PBS. Then 30 μm sections were permeabilized and blocked in 0.1 M Tris, containing 0.1% Triton X-100 (MilliporeSigma), 1% normal mouse serum, 1% normal rat serum, and 1% BSA (R&D Systems). Samples were stained for 2 hours at RT in a wet chamber with the appropriate antibodies, washed 3 times in PBS, and mounted in Fluoromount-G (Southern Biotech). Images were acquired using a TCS SP8 (Leica Microsystems) inverted confocal microscope, on a 40× 1.3 N/A oil or 40× 1.1 N/A water objective. Raw imaging data were processed using Imaris (Bitplane). Antibodies used in this study are in Supplemental Table 1.

### Iterative staining

Iterative staining of sections was performed as previously described (47, 48). Samples were prepared and stained as described above. Following acquisition, the coverslip was removed, and slides were washed 3 times in PBS to remove any mounting medium. Bleaching of the fluorochromes was achieved using a 1 mg/mL solution of lithium borohydride in water (Acros Organics) for 15 minutes at RT. The slides were then washed 3 times in PBS prior to staining with a different set of antibodies as described above. The process was repeated up to 5 times. Raw imaging data were processed using Imaris (Bitplane) using Hoechst or CD31 as fiducial for the alignment of subsequent images.

### Production of anti-OVA antibodies in mouse

Mice were immunized subcutaneously with 100 μL of OVA in incomplete Freund’s adjuvant (50 μg of OVA per immunization) on day 0 and day 14. On day 28, mice were culled via CO_2_, and blood was collected into serum separation tubes (Sarstedt) via cardiac puncture. After 30 minutes at room temperature, the tubes were centrifuged at 2,000*g* for 5 minutes at 4°C, and serum was collected for later IgG purification.

### IgG purification from serum

Serum from OVA-immunized mice was collected as previously described. IgG was purified using the Pierce Protein A IgG purification kit following the manufacturer’s protocol, using sterile PBS as binding/washing buffer; 0.1 M glycine with 150 mM NaCl, pH 2.5, as elution buffer; and 1 M Tris, pH 7, as neutralization buffer. IgG content was measured on a NanoDrop 2000 (Thermo Fisher Scientific). Amicon Ultra 15 mL centrifugal filters (Merck) were used to exchange buffer for sterile PBS, and the purified IgG solution was kept at –20°C until required.

### Nephrotoxic nephritis

Mice were given a single intravenous injection of 50 μL sheep anti-rat isolated glomerular basement membrane serum (ProbeTex) on day 0. Proteinuria was monitored daily using Uristix 11 from day 1, and the mice were euthanized for analysis on day 4.

### Cell collection and library preparation for single-cell sequencing

Cells were flow-sorted as previously described and centrifuged at 350*g* for 5 minutes at 4°C. Supernatant was discarded and the pellet resuspended in PBS 0.05% BSA. Cells were loaded at an appropriate concentration to enable recovery of 10,000 cells. A 10x Genomics single-cell 3′ v2 kit was used as per the manufacturer’s protocol. Libraries were produced using the manufacturer’s protocol and sequenced on a HiSeq 4000 (Illumina). The single-cell RNA-sequencing data are publicly available at NCBI Gene Expression Omnibus repository under the accession number GSE215272.

### Mouse single-cell data analysis and preprocessing

Single-cell gene expression data from cellranger output was analyzed using standard Seurat-inspired scanpy (v.1.4.5.post2) workflow (49, 50). Doublet detection was performed using scrublet (v0.2.1) (51) with adaptations as outlined before (52). Briefly, after scrublet was performed, the data were iteratively subclustered using standard Seurat-inspired scanpy (v.1.4.5.post2) workflow (49, 50), and a median scrublet score for each subcluster was computed. Median absolute deviation scores were computed from the cluster scrublet scores, and a 1-tailed *t* test was performed with Benjamini-Hochberg (BH) correction (53) applied. Cells with significantly outlying cluster scrublet scores (BH *P* < 0.1) were flagged as potential doublets. The data were then processed using scanpy with standard quality control steps; cells were filtered if number of genes was >2,500 or <200. Percentage mitochondrial content cutoff was set at <5%. Genes were retained if they were expressed by at least 3 cells. Gene counts for each cell were normalized to contain a total count equal to the median of total counts in cells before normalization. This led to a working data set of 3,654 cells. Highly variable genes were selected based on the following parameters: minimum and maximum mean expression ≥ 0.0125 and ≤ 3, respectively; minimum dispersion of genes = 0.5. The number of principal components used for neighborhood graph construction and dimensional reduction was set at 50. Batch correction was performed using bbknn with strains as the batch term and with all other parameters as per default settings (54). Clustering was performed using Leiden algorithm (55) with resolution set at 1.0. UMAP (v3.10.0) (56) was used for dimensional reduction and visualization, the minimum distance was set at 0.3, and all other parameters were as per default settings in scanpy.

### Human single-cell data analysis

Normalized single-cell data from normal and lupus nephritis human kidneys were downloaded (37, 38). The 2 data sets were integrated with ingest protocol in scanpy.

### Differential gene testing

Differential gene testing was performed using the Wilcoxon rank sum test implemented in scanpy’s rank_genes_groups module.

### Gene set testing

Gene set testing was performed using AUCell analysis tool (57). Gene sets from the respective studies were downloaded from ArrayExpress (YS versus HSC signature, E-MEXP-3510); MSigDB (58, 59); GO_FC_GAMMA_RECEPTOR_SIGNALING_PATHWAY, KEGG_FC_GAMMA_R_MEDIATED_PHAGOCYTOSIS, GSE7509_FCGRIIB_STIM_MONOCYTE_ DOWN and GSE7509_FCGRIIB_STIM_DC_ DOWN (60); interferon signatures (61); macrophage stimulation signatures (62); and opsonized immune complex signatures (63). Heatmaps were generated using the pheatmap R package.

### Cell type similarity assessment

We used a logistic regression approach to test for cell type similarity with Lyve1^+^/Lyve1^–^ macrophages (19). This is done with L2-regularized logistic regression (ridge regression) binomial model with the glmnet R package (64) (i.e., α parameter = 0). Models were trained on normalized gene expression data with 10-fold cross-validation to obtain the appropriate λ coefficient (lambda.1se; within 1 standard error from best model) for prediction. Genes were filtered for tissue-specific genes (19) prior to model training. Gene expression values were standardized in both the training and test sets. The average of 50 iterations was used for the final score.

### Trajectory analyses

Cell trajectory analyses were performed using slingshot (65) and tradeSeq (66). The cells were ordered based on the root node closest to clusters deemed to be composed of proliferating/dividing cells (group 1.6) or monocytes (group 2.1). Principal component analysis was used for learning the trajectory.

### TF enrichment analysis

TF and regulon enrichment was performed using pyscenic (67). The top 10 cluster-specific regulons were determined by calculating regulon specificity scores and significantly differently enriched regulons based on Wilcoxon rank sum tests between clusters of interest (group 1.4 vs. group 1.2 and group 2.3 vs. group 2.2).

### CellPhoneDB analysis

Normalized expression values from macrophage cell types found in this data set and the macrophage and human data sets were subjected to CellPhoneDB analysis (v2.0.0) (68). The minimum threshold was set at 30%, and results were considered statistically significant if *P* < 0.05.

### ST analysis

Analysis of Visium Spatial Gene Expression data was performed using Seurat v3.2.3 as per default methods. Prediction/label transfer was performed using the default SCTransform protocol. Correlation of molecules on the Visium data was performed by computing the correlation score across overlapping k-nearest neighborhoods of a spot based on spatial coordinates, followed by averaging across the neighborhoods. If any neighborhoods returned with no available values, due to uniform expression values across the entire neighborhood, the correlation value would not be returned and visualized as a gray spot. Only positive correlation values were retained for the analysis.

### Transcriptomic data visualization

#### Mean expression dot plot.

Size of circle indicates percentage of cells expressing genes, and increasing expression (scaled from 0 to 1) corresponds to increasing color gradient from white to blue (Figure 2B and Supplemental Figure 3F) or from purple, blue, green, to yellow (Figure 2E and Figure 3C).

#### Heatmap.

Row/column enrichment value is scaled from 0 to 1 and presented as an increasing gradient from white to blue (Figure 2D) or from purple, blue, green, to yellow (Figure 3D and Figure 8, B and C), which corresponds to increasing enrichment score.

#### CellPhoneDB.

The order of the receptor-ligand interactions corresponds to the order of the cell types, i.e., cell type A expressing molecule A interacts with cell type B expressing molecule B. Size of circles and color gradient correspond to the receptor-ligand interaction score, with purple, blue, green, to yellow for increasing values (Figure 5A and Figure 8F).

#### ST.

Expression of molecules/genes per spot are colored from increasing gradient from purple to green to yellow to red (Figure 2F and Figure 7B) or from white to red (Figure 3, E and F, and Figure 5B), which corresponds to increasing expression value.

### Statistics

Statistical analyses were performed in R, python, or Prism (GraphPad) where appropriate. For the single-cell data analyses, in general, nonparametric tests were used and stated in the corresponding figure legends. BH or Bonferroni post hoc correction procedure was applied for multiple testing correction, and adjusted *P* < 0.05 was considered statistically significant. Dot plots represent mean ± SD.

### Study approval

All animal procedures were conducted in accordance with the United Kingdom Animals (Scientific Procedures) Act of 1986. In the United States, all animal study protocols were approved by the animal care and use committee of the NIAMS/NIH, listed on animal study protocol AO14-01–01, and in agreement with Animal Research Advisory Committee guidelines (3.18.1).

Kidney biopsies from 3 patients with a diagnosis of lupus nephritis (LN III/IV/V each) and 3 healthy portions of kidneys obtained during nephrectomy of patients with a diagnosis of renal cell carcinoma were obtained through NIH protocol 94-AR-0066. All tissue samples were obtained in accordance with the NIH–Clinical Center Ethics Committee procedures, and all patients provided written informed consent.

## Author contributions

Research study design was contributed by NR, MJK, RMS, and MRC. Conducting experiments was contributed by NR, KWL, EPM, JRF, and AP. Data analysis was contributed by NR and ZKT. KRB, ET, FR, TH, and TJ provided reagents. NR (first) and ZKT (second) are co–first authors based on their degrees of contribution.

## Supplementary Material

Supplemental data

Supplemental table 1

## Version 1 11/08/2022

Electronic publication

## Version 2 11/10/2022

Authorship note changed and corrected from MR to NR

## References

[1] Guilliams M (2014). Dendritic cells, monocytes and macrophages: a unified nomenclature based on ontogeny. Nat Rev Immunol.

[2] Rahman A, Isenberg DA (2008). Systemic lupus erythematosus. N Engl J Med.

[3] Clatworthy MR, Smith KG (2007). B cells in glomerulonephritis: focus on lupus nephritis. Semin Immunopathol.

[4] Smith KGC, Clatworthy MR (2010). FcgammaRIIB in autoimmunity and infection: evolutionary and therapeutic implications. Nat Rev Immunol.

[5] Floto RA (2005). Loss of function of a lupus-associated FcgammaRIIb polymorphism through exclusion from lipid rafts. Nat Med.

[6] Jing C (2020). Macrophage metabolic reprogramming presents a therapeutic target in lupus nephritis. Proc Natl Acad Sci U S A.

[7] Willcocks LC (2009). Low-affinity Fcgamma receptors, autoimmunity and infection. Expert Rev Mol Med.

[8] Cassese G (2001). Inflamed kidneys of NZB / W mice are a major site for the homeostasis of plasma cells. Eur J Immunol.

[9] Espeli M (2010). Local renal autoantibody production in lupus nephritis. J Am Soc Nephrol.

[10] Smith RM (2010). Biological therapy for lupus nephritis-tribulations and trials. Nat Rev Rheumatol.

[11] Schulz C (2012). A lineage of myeloid cells independent of Myb and hematopoietic stem cells. Science.

[12] Ginhoux F, Guilliams M (2016). Tissue-resident macrophage ontogeny and homeostasis. Immunity.

[13] Liu Z (2019). Fate mapping via Ms4a3-expression history traces monocyte-derived cells. Cell.

[14] Sheng J (2015). Most tissue-resident macrophages except microglia are derived from fetal hematopoietic stem cells. Immunity.

[15] Lever JM (2018). Parabiosis reveals leukocyte dynamics in the kidney. Lab Invest.

[16] Salei N (2020). The kidney contains ontogenetically distinct dendritic cell and macrophage subtypes throughout development that differ in their inflammatory properties. J Am Soc Nephrol.

[17] Liu F (2020). Distinct fate, dynamics and niches of renal macrophages of bone marrow or embryonic origins. Nat Commun.

[18] Mass E (2016). Specification of tissue-resident macrophages during organogenesis. Science.

[19] Chakarov S (2019). Two distinct interstitial macrophage populations coexist across tissues in specific subtissular niches. Science.

[20] Stamatiades EG (2016). Immune monitoring of trans-endothelial transport by kidney-resident macrophages. Cell.

[21] Cairns SA (1980). The value of three immune complex assays in the management of systemic lupus erythematosus: an assessment of immune complex levels, size and immunochemical properties in relation to disease activity and manifestations. Clin Exp Immunol.

[22] Tung KS (1981). Study of circulating immune complex size in systemic lupus erythematosus. Clin Exp Immunol.

[23] Paludan SR, Bowie AG (2013). Immune sensing of DNA. Immunity.

[24] Ablasser A, Hur S (2020). Regulation of cGAS- and RLR-mediated immunity to nucleic acids. Nat Immunol.

[25] Adachi M (1993). Aberrant transcription caused by the insertion of an early transposable element in an intron of the Fas antigen gene of lpr mice. Proc Natl Acad Sci U S A.

[26] Park J (2018). Single-cell transcriptomics of the mouse kidney reveals potential cellular targets of kidney disease. Science.

[27] Soucie EL (2016). Lineage-specific enhancers activate self-renewal genes in macrophages and embryonic stem cells. Science.

[28] Lawrence T, Natoli G (2011). Transcriptional regulation of macrophage polarization: enabling diversity with identity. Nat Rev Immunol.

[29] Hu X, Ivashkiv LB (2009). Cross-regulation of signaling pathways by interferon-gamma: implications for immune responses and autoimmune diseases. Immunity.

[30] Niemand C (2003). Activation of STAT3 by IL-6 and IL-10 in primary human macrophages is differentially modulated by suppressor of cytokine signaling 3. J Immunol.

[31] Tridandapani S (2002). Src homology 2 domain-containing inositol polyphosphate phosphatase regulates NF-kappa B-mediated gene transcription by phagocytic Fc gamma Rs in human myeloid cells. J Immunol.

[32] Murphy DB (1992). Monoclonal antibody detection of a major self peptide. MHC class II complex. J Immunol.

[33] Vento-Tormo R (2018). Single-cell reconstruction of the early maternal-fetal interface in humans. Nature.

[34] Migeotte I (2005). Identification and characterization of an endogenous chemotactic ligand specific for FPRL2. J Exp Med.

[35] Li M (2009). Endogenous CD100 promotes glomerular injury and macrophage recruitment in experimental crescentic glomerulonephritis. Immunology.

[36] Ishida I (2003). Involvement of CD100, a lymphocyte semaphorin, in the activation of the human immune system via CD72: implications for the regulation of immune and inflammatory responses. Int Immunol.

[37] Stewart BJ (2019). Spatiotemporal immune zonation of the human kidney. Science.

[38] Arazi A (2019). The immune cell landscape in kidneys of patients with lupus nephritis. Nat Immunol.

[39] Bentham J (2015). Genetic association analyses implicate aberrant regulation of innate and adaptive immunity genes in the pathogenesis of systemic lupus erythematosus. Nat Genet.

[40] Zhang YM (2017). Association of the IKZF1 5’ UTR variant rs1456896 with lupus nephritis in a northern Han Chinese population. Scand J Rheumatol.

[41] Mackay F (2003). BAFF AND APRIL: a tutorial on B cell survival. Annu Rev Immunol.

[42] Avery DT (2003). BAFF selectively enhances the survival of plasmablasts generated from human memory B cells. J Clin Invest.

[43] Ramanujam M (2010). Selective blockade of BAFF for the prevention and treatment of systemic lupus erythematosus nephritis in NZM2410 mice. Arthritis Rheum.

[44] Navarra SV (2011). Efficacy and safety of belimumab in patients with active systemic lupus erythematosus: a randomised, placebo-controlled, phase 3 trial. Lancet.

[45] Dooley MA (2013). Effect of belimumab treatment on renal outcomes: results from the phase 3 belimumab clinical trials in patients with SLE. Lupus.

[46] Wu HJ, Bondada S (2002). Positive and negative roles of CD72 in B cell function. Immunol Res.

[47] Radtke AJ (2022). IBEX: an iterative immunolabeling and chemical bleaching method for high-content imaging of diverse tissues. Nat Protoc.

[48] Radtke AJ (2020). IBEX: a versatile multiplex optical imaging approach for deep phenotyping and spatial analysis of cells in complex tissues. Proc Natl Acad Sci U S A.

[49] Wolf FA (2018). SCANPY: large-scale single-cell gene expression data analysis. Genome Biol.

[50] Stuart T (2019). Comprehensive integration of single-cell data. Cell.

[51] Wolock SL (2019). Scrublet: computational identification of cell doublets in single-cell transcriptomic data. Cell Syst.

[52] Popescu DM (2019). Decoding human fetal liver haematopoiesis. Nature.

[53] Benjamini Y, Hochberg Y (1995). Controlling the false discovery rate: a practical and powerful approach to multiple testing. J R Stat Soc Series B Stat Methodol.

[54] Polanski K (2020). BBKNN: fast batch alignment of single cell transcriptomes. Bioinformatics.

[55] Traag VA (2019). From Louvain to Leiden: guaranteeing well-connected communities. Sci Rep.

[56] https://arxiv.org/abs/1802.03426.

[57] Aibar S (2017). SCENIC: single-cell regulatory network inference and clustering. Nat Methods.

[58] Subramanian A (2005). Gene set enrichment analysis: a knowledge-based approach for interpreting genome-wide expression profiles. Proc Natl Acad Sci U S A.

[59] Liberzon A (2011). Molecular signatures database (MSigDB) 3.0. Bioinformatics.

[60] Dhodapkar KM (2007). Selective blockade of the inhibitory Fcgamma receptor (FcgammaRIIB) in human dendritic cells and monocytes induces a type I interferon response program. J Exp Med.

[61] Baechler EC (2003). Interferon-inducible gene expression signature in peripheral blood cells of patients with severe lupus. Proc Natl Acad Sci U S A.

[62] Xue J (2014). Transcriptome-based network analysis reveals a spectrum model of human macrophage activation. Immunity.

[63] Castro-Dopico T (2019). Anti-commensal IgG drives intestinal inflammation and type 17 immunity in ulcerative colitis. Immunity.

[64] Friedman JH (2010). Regularization paths for generalized linear models via coordinate descent. J Stat Softw.

[65] Street K (2018). Slingshot: cell lineage and pseudotime inference for single-cell transcriptomics. BMC Genomics.

[66] Van den Berge K (2020). Trajectory-based differential expression analysis for single-cell sequencing data. Nat Commun.

[67] Van de Sande B (2020). A scalable SCENIC workflow for single-cell gene regulatory network analysis. Nat Protoc.

[68] Efremova M (2020). CellPhoneDB: inferring cell-cell communication from combined expression of multi-subunit ligand-receptor complexes. Nat Protoc.

