# Distinct pathogenic roles for resident and monocyte-derived macrophages in lupus nephritis

**DOI:** 10.1172/jci.insight.171762

**Published:** 2023-05-22

**Authors:** Nathan Richoz, Zewen K. Tuong, Kevin W. Loudon, Eduardo Patiño-Martínez, John R. Ferdinand, Anaïs Portet, Kathleen R. Bashant, Emeline Thevenon, Francesca Rucci, Thomas Hoyler, Tobias Junt, Mariana J. Kaplan, Richard M. Siegel, Menna R. Clatworthy

Original citation: *JCI Insight*. 2022;7(21):e159751. https://doi.org/10.1172/jci.insight.159751

Citation for this corrigendum: *JCI Insight*. 2023;8(10):e171762. https://doi.org/10.1172/jci.insight.171762

Jorge Romo-Tena’s name was inadvertently omitted from the author list and *Author contributions* section. The correct author and affiliations lists and sentence from the *Author contributions* section are below. The HTML and PDF versions have been updated online.

Nathan Richoz,^1,2,3^ Zewen K. Tuong,^1,2,4^ Kevin W. Loudon,^1,2^ Eduardo Patiño-Martínez,^3^ John R. Ferdinand,^1,2^ Anaïs Portet,^1,2^ Jorge Romo-Tena,^3^ Kathleen R. Bashant,^3^ Emeline Thevenon,^5^ Francesca Rucci,^5^ Thomas Hoyler,^5^ Tobias Junt,^5^ Mariana J. Kaplan,^3^ Richard M. Siegel,^3,5^ Menna R. Clatworthy^1,2,4^

^1^Molecular Immunity Unit, University of Cambridge Department of Medicine, Medical Research Council Laboratory of Molecular Biology, Cambridge, United Kingdom. ^2^Cambridge Institute of Therapeutic Immunology and Infectious Diseases, Department of Medicine, University of Cambridge School of Clinical Medicine, Cambridge, United Kingdom. ^3^National Institute of Arthritis and Musculoskeletal and Skin Diseases, National Institutes of Health, Bethesda, Maryland, USA. ^4^Cellular Genetics Programme, Wellcome Sanger Institute, Hinxton, United Kingdom. ^5^Novartis Institutes for BioMedical Research, Basel, Switzerland.

Conducting experiments was contributed by NR, KWL, EPM, JRF, AP, and JRT.

## Version 1 05/22/2023

Electronic publication

