# Supplementary material for: Distinct pathogenic roles for resident and monocyte-derived macrophages in lupus nephritis
Source: JCI Insight. 2022 Nov 8;7(21):e159751. doi: 10.1172/jci.insight.159751 (PMC9675473; doi:10.1172/jci.insight.159751)
Supplement: Supplemental data [file jciinsight-7-159751-s308.pdf]

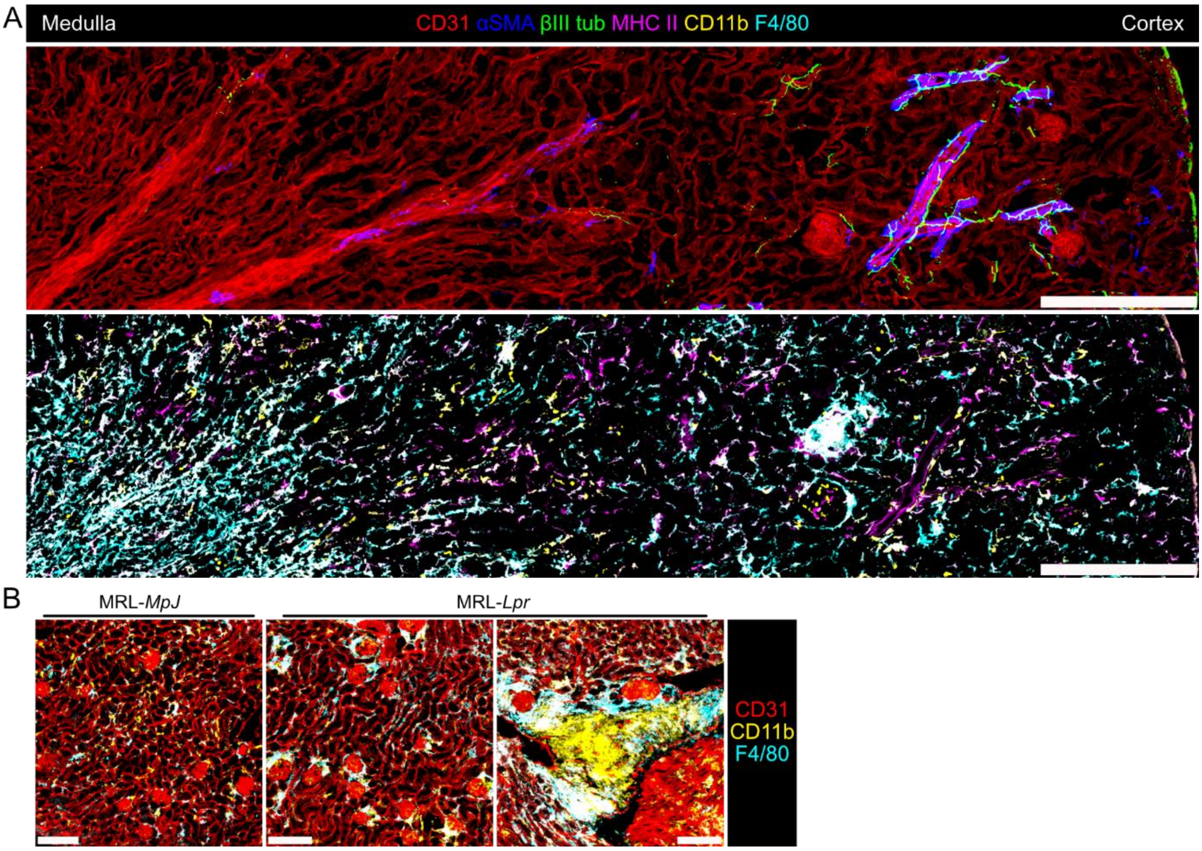


**Supplemental figure 1: Macrophage populations expand with inflammation.** (**A**) Representative iterative staining (n = 3) on murine C57BL/6 kidney showing MNP distribution throughout the tissue through expression of F4/80 (cyan), CD11b (yellow) and MHC II (magenta) (bottom) relative to blood vessels (CD31, red) and surrounding smooth muscles (αSMA, blue) and nerves (βIII tubulin, green) (top). Scale bar = 350µm (**B**) Representative confocal microscopy (n = 3 per group) showing MNP distribution in kidneys from 18-week-old MRL-*MpJ* (left) and MRL-*Lpr* (middle and right) mice through expression of F4/80 (cyan) and CD11b (yellow) relative to blood vessels (CD31, red). Scale bar = 150µm.

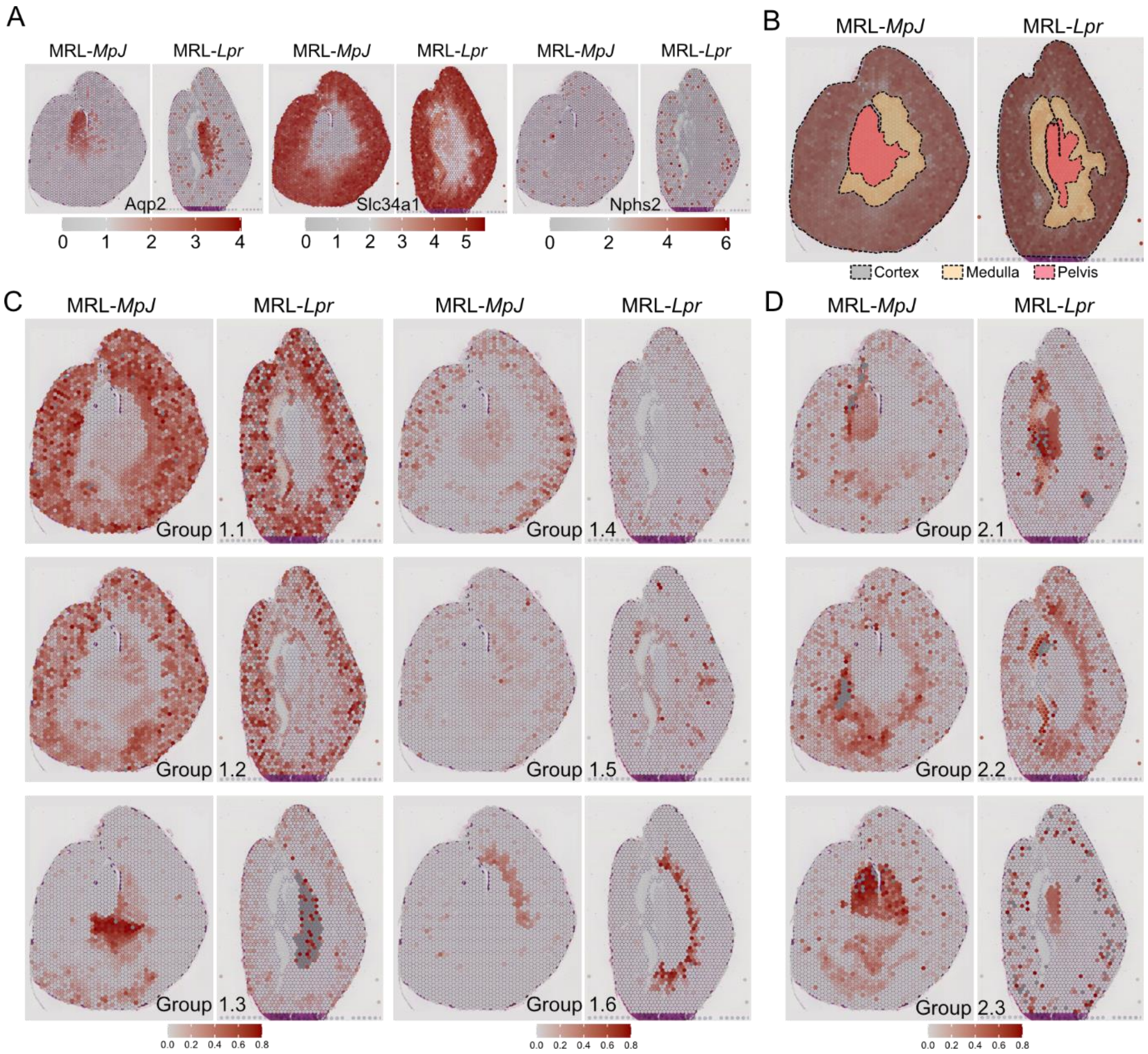


**Supplemental figure 2: Spatial distribution of kidney macrophage populations.** (**A**) Spatial expression of markers used to delineate the anatomical regions in Visium Spatial Gene Expression data of MRL-*MpJ* and MRL-*Lpr* kidney sections (Aqp2 – pelvis; Slc34a1 = proximal tubules and Nphs2 – glomeruli). (**B**) Delineation of anatomical regions in MRL-*MpJ* and MRL-*Lpr* visium kidney sections based on (A). (**C**) Spatial transcriptomics of Group 1 macrophages signatures in MRL-*MpJ* and MRL-*Lpr* murine kidneys with annotated scRNAseq data above. Each spot / voxel denotes a prediction score of 0 – 1 for the location of each of the macrophage subgroups. (**D**) Spatial transcriptomics of Group 2 macrophages signatures in MRL-*MpJ* and MRL-*Lpr* murine kidneys with annotated scRNAseq data above. Each spot / voxel denotes a prediction score of 0 – 1 for the location of each of the macrophage subgroups.

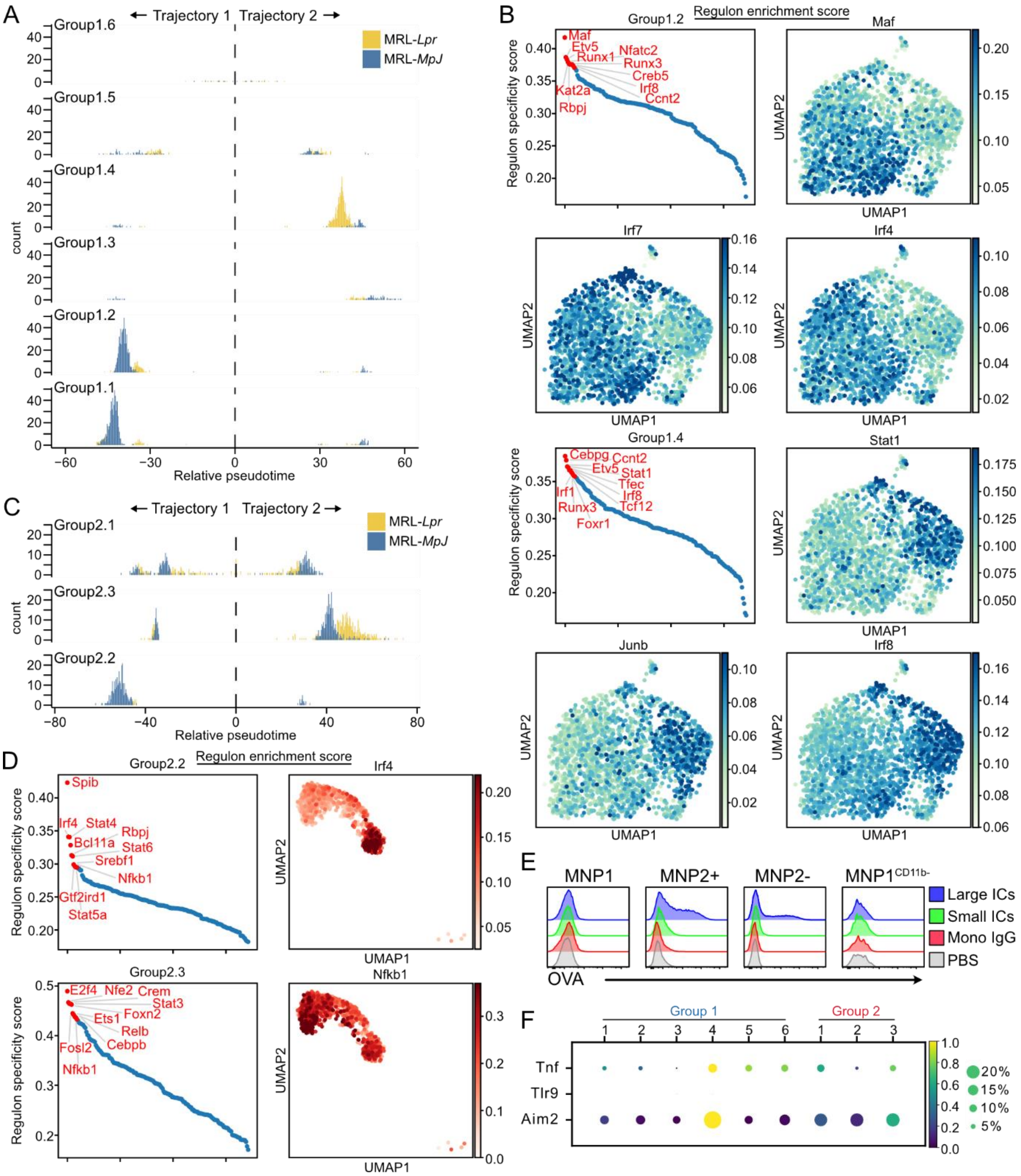


**Supplemental figure 3: Trajectory and regulon analysis of kidney macrophages.** (**A**) Proportion of cells from MRL-*MpJ* (blue) or MRL-*Lpr* (yellow) from each Group 1 cluster distributed along pseudotime scale. (**B**) (Top) Top ranked regulons in trajectory 1 for Group 1 (left) and enrichment UMAP of regulon activity of Maf, Irf7 and Irf4 in Group 1.2 trajectory (trajectory 1). (Bottom) Top ranked regulons in trajectory 2 for Group 1 (left) and enrichment UMAP of regulon activity of Stat1, Junb and Irf8 in Group 1.4 trajectory (trajectory 2). (**C**) Proportion of cells from MRL-*MpJ* (blue) or MRL-*Lpr* (yellow) from each Group 2 cluster distributed along pseudotime scale. (**D**) (Top left) Top ranked regulons in trajectory 1 for Group 2. (top right) Enrichment UMAP of regulon activity of Irf4 in Group 2.2 trajectory

(trajectory 1). (Bottom left) Top ranked regulons in trajectory 2 for Group 2. (Bottom right) Enrichment UMAP of regulon activity of Nfkb1 in Group 2.3 trajectory (trajectory 2). (**E**) Uptake of free and immune complexed AF647-OVA by kidney MNPs in vivo 2h following intravenous injection in C57BL/6 mice. 5:1 (large) and 1:1 (small) molar ratios of mouse IgGs:OVA were used. (**F**) Mean expression dot plot of genes Tnf, Tlr9 and Aim2.

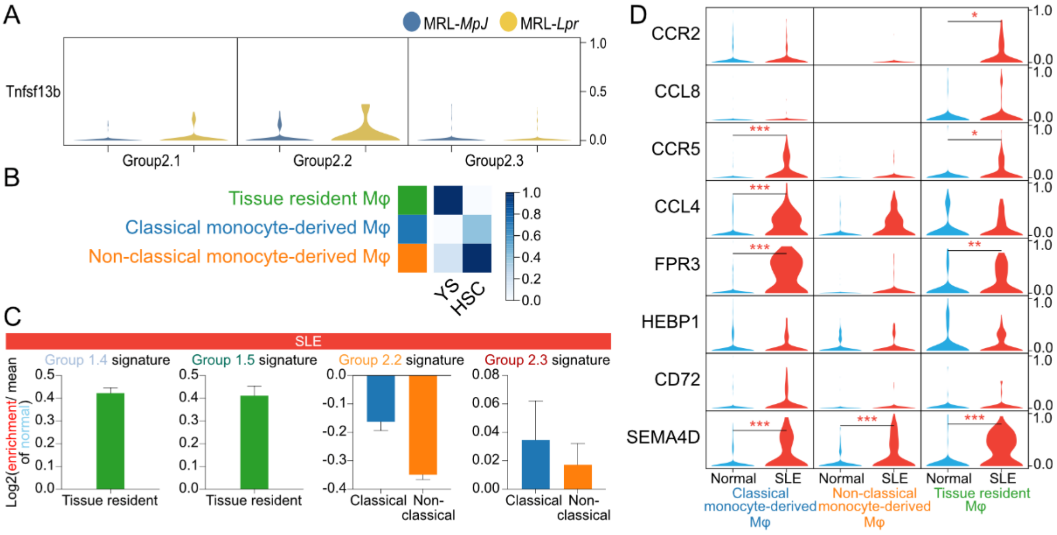


**Supplemental figure 4: Kidney macrophages in human SLE.** (**A**) Violin plot of Tnfsf13b (BAFF) in Group 2 clusters. (**B**) Heatmap of mean AUCell enrichment of F4/80hi/lo gene sets, corresponding to yolk sac (YS) vs hematopoetic stem-cell (HSC) lineage. Row enrichment value is scaled from 0 to 1 and presented as an increasing gradient from white to blue which corresponds to increasing enrichment score. (**C**) Bar charts showing relative log2 difference of AUCell enrichment of mouse Group1 and Group2 macrophage clusters in SLE compared to normal (mean of normal enrichment used as reference). (**D**) Violin plot of molecules, with significant interactions found in mouse macrophage in (A), in human macrophage clusters. Expression value is scaled from 0 to 1 across cell clusters. Significance was calculated using Wilcoxon rank sum test with BH post-test applied where *p<0.05; ***p<0.001. Colour of the p-value indicates which group has a higher value (red = SLE, blue = normal).

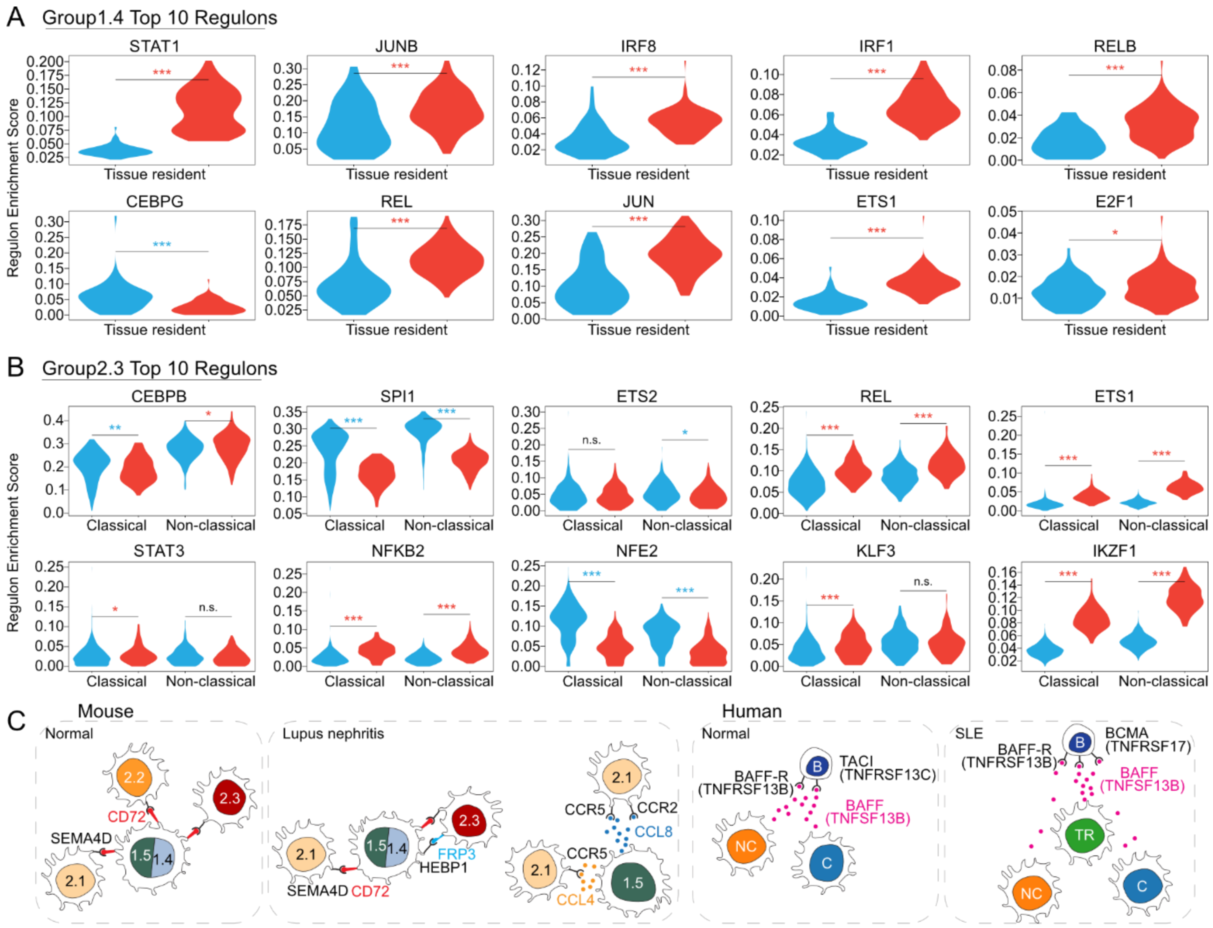


**Supplemental figure 5: Top regulons in kidney macrophages.** (**A**) Violin plot of regulon enrichment scores in human tissue resident macrophages split by normal (blue) or SLE (red) for Group1.4-related top 10 transcription factors. Significance was calculated using Wilcoxon rank sum test with BH post-test applied where *p<0.05; ***p<0.001. Colour of the p-value indicates which group has a higher value (red = SLE, blue = normal). (**B**) Violin plot of regulon enrichment scores in human monocyte-derived macrophages split by normal (blue) or SLE (red) for Group2.3-related top 10 transcription factors. Significance was calculated using Wilcoxon rank sum test with BH post-test applied where *p<0.05; ***p<0.001. Colour of the p-value indicates which group has a higher value (red = SLE, blue = normal). (**C**) Summary illustration of macrophage interactions in mouse and human kidneys.
